# The Parkinson’s Disease Protein LRRK2 Interacts with the GARP Complex to Promote Retrograde Transport to the *trans*-Golgi Network

**DOI:** 10.1016/j.celrep.2020.107614

**Published:** 2020-05-05

**Authors:** Alexandra Beilina, Luis Bonet-Ponce, Ravindran Kumaran, Jennifer J. Kordich, Morié Ishida, Adamantios Mamais, Alice Kaganovich, Sara Saez-Atienzar, David C. Gershlick, Dorien A. Roosen, Laura Pellegrini, Vlad Malkov, Matthew J. Fell, Kirsten Harvey, Juan S. Bonifacino, Darren J. Moore, Mark R. Cookson

**Affiliations:** 1 Laboratory of Neurogenetics, National Institute on Aging, National Institutes of Health, Bethesda, MD 20814, USA; 2 Center for Neurodegenerative Science, Van Andel Research Institute, Grand Rapids, MI 49503, USA; 3 Cell Biology and Neurobiology Branch, Eunice Kennedy Shriver National Institute of Child Health and Human Development, National Institutes of Health, Bethesda, MD 20814, USA; 4 School of Pharmacy, University of Reading, Whiteknights, Reading, RG6 6AP, UK; 5 Department of Pharmacology, UCL School of Pharmacy, University College London, 29-39 Brunswick Square, London, WC1N 1AX, UK; 6 Merck & Co., Inc., 33 Avenue Louis Pasteur, Boston, MA 02115, USA; 7 These authors contributed equally; 8 Lead Contact

## Abstract

Mutations in Leucine-rich repeat kinase 2 (LRRK2) cause Parkinson’s disease (PD). However, the precise function of LRRK2 remains unclear. We report an interaction between LRRK2 and VPS52, a subunit of the Golgi-associated retrograde protein (GARP) complex that identifies a function of LRRK2 in regulating membrane fusion at the *trans*-Golgi network (TGN). At the TGN, LRRK2 further interacts with the Golgi SNAREs VAMP4 and Syntaxin-6 and acts as a scaffolding platform that stabilizes the GARP-SNAREs complex formation. Therefore, LRRK2 influences both retrograde and post-Golgi trafficking pathways in a manner dependent on its GTP binding and kinase activity. This action is exaggerated by mutations associated with Parkinson’s disease and can be blocked by kinase inhibitors. Disruption of GARP sensitizes dopamine neurons to mutant LRRK2 toxicity in *C. elegans*, showing that these pathways are interlinked *in vivo* and suggesting a link in PD.

## In Brief

Mutations in *LRRK2* are linked to Parkinson’s disease. However, the cellular role of LRRK2 remains elusive. Using a combination of proteomics and imaging techniques, Beilina et al. describe a function of LRRK2 mediating endosome-TGN transport by scaffolding the GARP:Syntaxin-6 interaction, suggesting a connection between GARP and LRRK2 in Parkinson’s disease.

## Graphical Abstract

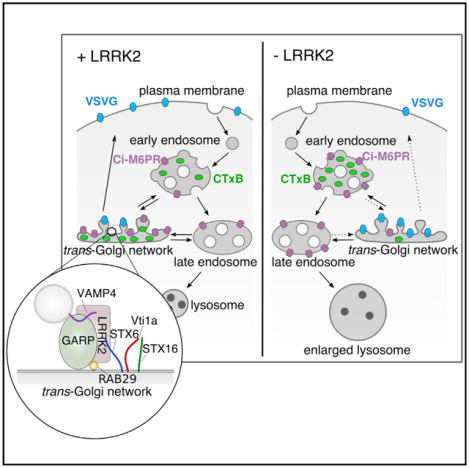

## INTRODUCTION

Mutations in the *LRRK2* gene are a major cause of Parkinson’s disease (PD), a common age-dependent neurodegenerative disorder characterized by neuronal damage in multiple brain regions and consequent motor defects (Cookson, 2010). Genome-wide association studies (GWASs) have also nominated the LRRK2 locus as a risk factor for PD (Simón-Sánchez et al., 2009).

LRRK2 encodes a large protein with multiple protein-protein interaction domains and two enzymatic domains: kinase and GTPase. The PD-associated kinase domain mutations (G2019S and I2020T) were reported to increase kinase activity both *in vitro* and *in vivo*, and GTPase-deficient mutations (R1441C/G/H and Y1699C) were reported to enhance kinase activity *in vivo* (Greggio et al., 2006; Lewis et al., 2007; Steger et al., 2016). Unfortunately, the normal function of LRRK2 or how pathological gain of function mutations lead to neurotoxicity are yet to be elucidated.

Multiple lines of evidence suggest that LRRK2 plays some undefined role in the endo-lysosomal system. LRRK2 is present at multiple intracellular membranes (Alegre-Abarrategui et al., 2009; Biskup et al., 2006), possibly related to its ability to bind (Beilina et al., 2014; Dodson et al., 2012; MacLeod et al., 2013) and phosphorylate (Ito et al., 2016; Steger et al., 2016; Thirstrup et al., 2017; Yun et al., 2015) RAB proteins.

Although expression of LRRK2 mutants or inhibition of its normal function affects the lysosome-autophagy system in various contexts (Bravo-San Pedro et al., 2013; Dodson et al., 2014; Gómez-Suaga et al., 2012; Manzoni et al., 2013; Soukup et al., 2016; Tong et al., 2012), the question of what LRRK2 does at membranes leading to altered regulation of the endo-lysosomal system has not been resolved.

We and others have previously shown that unbiased surveys of protein-protein interactions can be useful to nominate functions of LRRK2. Specifically, a physical interaction between LRRK2 and the small GTPase RAB29 (also known as RAB7L1) is sufficient to recruit LRRK2 to the *trans*-Golgi network (TGN), influencing Golgi morphology (Beilina et al., 2014; MacLeod et al., 2013). Subsequently, it has been shown that RAB29 is both a substrate and activator of LRRK2 at the TGN (Liu et al., 2018; Purlyte et al., 2018). Here, we extend this approach to reveal a protein interaction between LRRK2 and vacuolar protein sorting (VPS) proteins, including components of the Golgi-associated retrograde protein (GARP) complex and endosome-associated recycling protein (EARP) complexes (Bonifacino and Hierro, 2011). We define the effects of LRRK2 and its interacting partners, particularly RAB29 and VPS52, on intracellular trafficking pathways. LRRK2 scaffolds the interaction between the GARP complex and the Golgi SNAREs, promoting TGN retrograde transport. As a consequence, LRRK2 regulates CI-M6PR trafficking, along with its cargo cathepsin D. These results strengthen the concept that the etiopathogenesis of PD is circumscribed by disrupted trafficking dynamics and identify potential therapeutic approaches in LRRK2 PD.

## RESULTS

### Sequential Screening Demonstrates that LRRK2 and Interacting Partners Form Part of an Extended Protein Interaction Network

**Figure 1.**
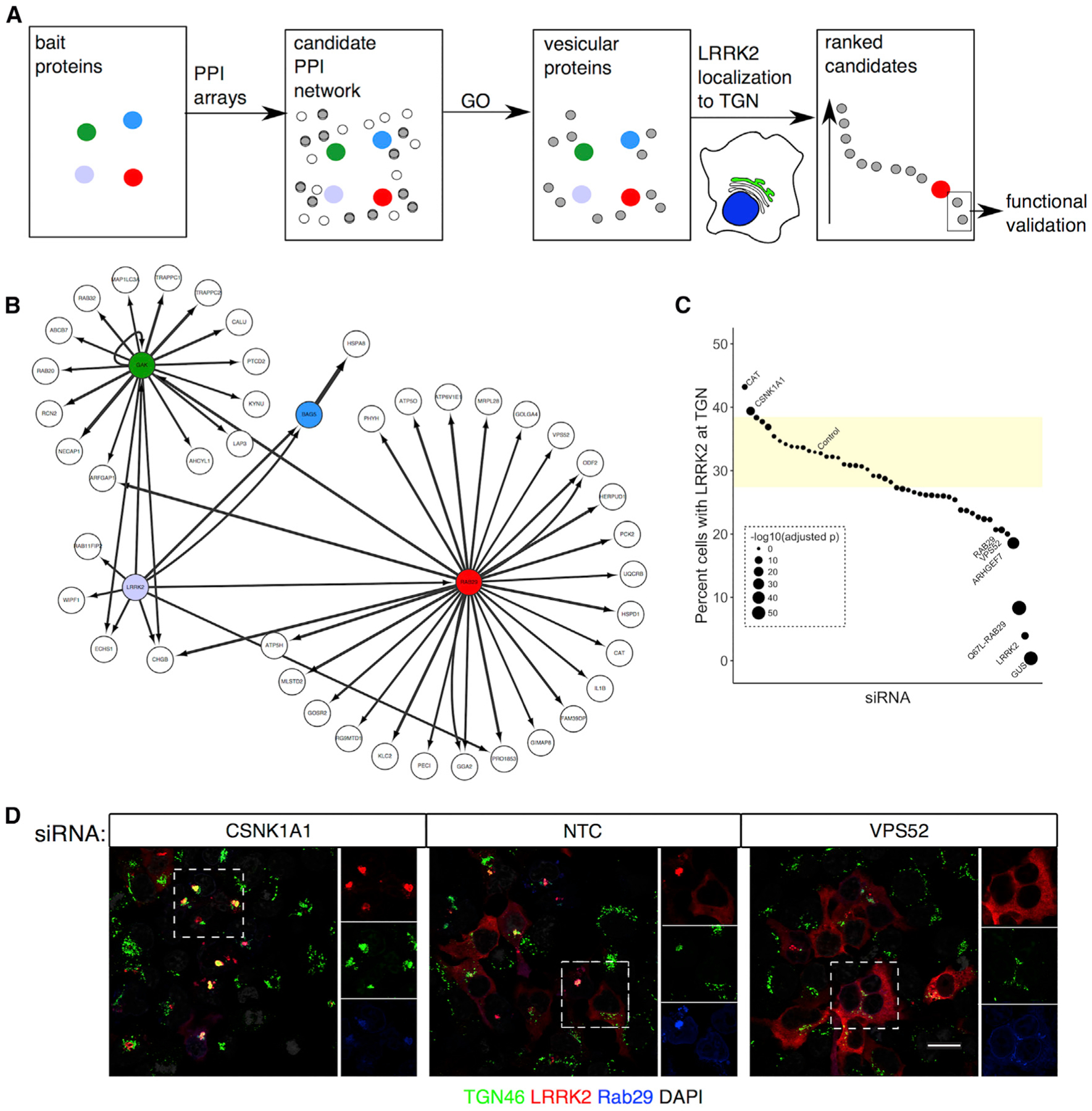
Sequential Screening Identifies VPS52 as a Physical and Functional Interactor of LRRK2 (A) Outline of the overall approach to screen for interactions of a LRRK2 complex. (B) The vesicle-associated network. Subnetwork of candidate protein-protein interactors generated by protein arrays for bait proteins LRRK2 (light blue), RAB29 (red), BAG5 (blue), and GAK (green), with VPS52 highlighted with a red outline. Arrows are sized by the normalized interaction strength (Z) from the protein arrays. (C) siRNA screen for functional effectors of TGN recruitment. Candidates from (B) were screened using the percentage of cells for which LRRK2 was recruited to the TGN after co-transfection with RAB29 (vertical axis; mean of n = 6 wells per siRNA). Points are sized by the Bonferroni-adjusted p value compared with the WT LRRK2 co-transfected with WT RAB29 and treated with non-targeting siRNA control (NTC_WT) from all plates used (n = 48 wells total; t test with Welch’s correction for unequal variance). The shaded area indicates 2 SDs from the mean of the reference WT LRRK2-transfected samples treated with a non-targeting control siRNA (NTC). These reference data were not significantly different from a normal distribution (Shapiro-Wilk test p = 0.8991) (D) Confirmation by confocal imaging. Confocal images of cells transfected with WT LRRK2 and WT RAB29, treated with indicated siRNA and stained for LRRK2, RAB29, and the TGN marker (TGN46).

We have previously used protein-protein interaction arrays to identify RAB29, GAK, and BAG5 as protein interactors of LRRK2 and demonstrated that the complex formed by these proteins is recruited to the TGN (Beilina et al., 2014). We hypothesized that these proteins could represent part of an extended interaction network and set out to further identify additional functional partners. To this end, we used a sequential screening approach consisting of, first, identification of protein-protein interactions for LRRK2, RAB29, BAG5, and GAK; second, classification of the interacting proteins by Gene Ontology (GO); and finally, their functional validation using a LRRK2-relevant assay of recruitment to the TGN (Figure 1A).

We purified the previously validated interactors of LRRK2—BAG5, GAK, and RAB29—from mammalian cells and performed protein-protein interaction arrays for each of them (Table S1). A protein interaction network based on direct interactions between the different proteins was constructed, and we found a number of common protein interactions for each probed protein (Figure S1A). Additionally, unique protein interactions were also found for each probed protein (Figures S1B–S1D).

The candidate network is large, which is consistent with prior computational estimates of the LRRK2 interactome (Manzoni et al., 2015; Tomkins et al., 2018). It is not clear, however, if there is a single large complex or multiple distinct complexes with different functions embedded within the network. We therefore looked for enrichment for GO terms within the overall network. Two noted enrichments that were consistent with prior analyses were “intracellular membrane bound organelle” and “cytoskeleton” (Figure S1E). Given our prior observation that LRRK2, RAB29, and GAK are recruited to the TGN, we next focused on the set of protein interactions identified by the GO term “membrane bound organelle” (Figure 1B), a set of 46 candidate interactors.

We next asked whether these proteins were functionally important for LRRK2 in cells. Specifically, we used our previously reported observation that overexpression of RAB29 (a small GTPase already present at the TGN) promotes the localization of LRRK2 to the TGN (Beilina et al., 2014; Chia et al., 2014). LRRK2 in cells shows predominantly cytoplasmic localization and enhanced RAB29 expression promotes LRRK2 recruitment to the TGN, as knockdown of overexpressed RAB29 reverses LRRK2 recruitment. Furthermore, GTPase-deficient and GTP-binding-deficient mutants of RAB29 cannot re-localize LRRK2 to TGN. Although the effect of knockdown of overexpressed RAB29 is an expected result, and the lack of effect of GTP-binding-deficient RAB29 is consistent with prior data, we used these controls to calibrate an objective morphological assay for LRRK2/RAB29 relocalization to the TGN on the basis of an automated microscopy platform. Screening small interfering RNA (siRNA) against each of the endogenous genes from the membrane organelle subnetwork confirmed that GAK, RAB29, and ArhGEF7 are each required for RAB29-dependent co-localization of LRRK2 with the TGN marker TGN46 (Figure 1C; Table S2). Additionally, we found that knockdown of VPS52, identified by our protein arrays as an interactor of RAB29, also resulted in the loss of LRRK2/Rab29 translocation to the TGN (Figure 1C). This was further validated using confocal microscopy (Figure 1D). These results show that VPS52 is a functional interactor of LRRK2 and RAB29 at the TGN.

### LRRK2 and RAB29 Interact with the GARP and EARP Complexes

**Figure 2.**
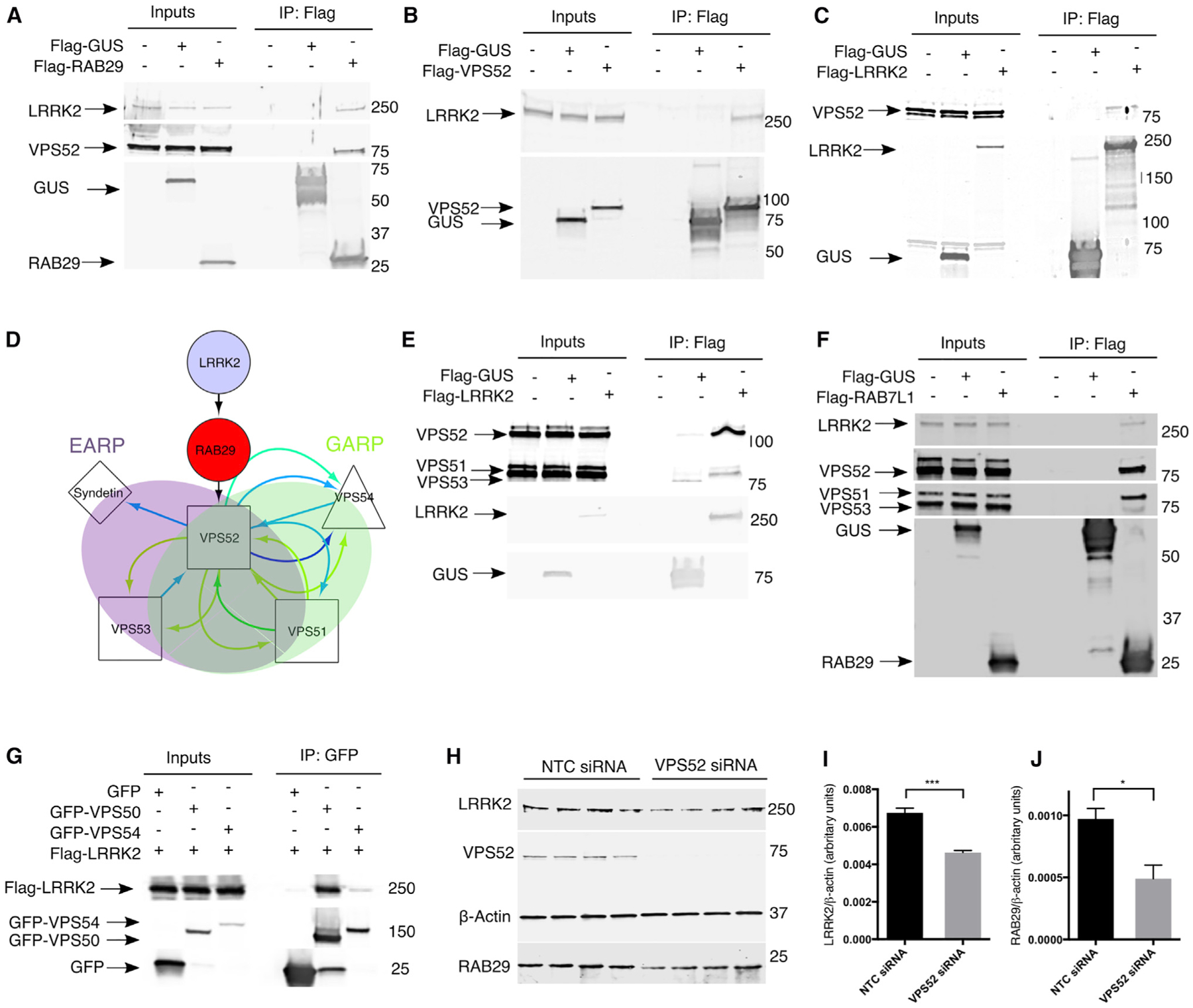
Validation of Interactions among LRRK2, RAB29, and the VPS52 Complex (A) RAB29 interacts with endogenous LRRK2 and VPS52. HEK293FT cells were either mock transfected or transfected with FLAG-tagged *E. coli* beta-glucuronidase (GUS) as negative controls or with FLAG-RAB29. Protein extracts were subjected to immunoprecipitation (IP) with anti-FLAG antibodies and immunoblotted for endogenous LRRK2, VPS52, and FLAG-protein baits. (B) VPS52 interacts with endogenous LRRK2. HEK293FT cells were either mock transfected or transfected with GUS or with FLAG-VPS52. Protein extracts were subjected to IP with anti-FLAG antibodies and immunoblotted for endogenous LRRK2 and FLAG-protein baits. (C) LRRK2 interacts with endogenous VPS52. HEK293FT cells were either mock transfected or transfected with GUS or with FLAG-LRRK2. Protein extracts were subjected to IP with anti-FLAG antibodies and immunoblotted for endogenous VPS52 and FLAG-protein baits. (D) Schematic of the EARP and GARP. Subnetwork of LRRK2, RAB29 VPS52 extended to known interactors of the latter protein. (E) LRRK2 interacts with endogenous GARP/EARP. HEK293FT cells were either mock transfected or transfected with GUS or with FLAG-LRRK2. Protein extracts were subjected to IP with anti-FLAG antibodies and immunoblotted for (from top to bottom) endogenous VPS52, VPS51, and VPS53 and FLAG-protein baits. (F) RAB29 interacts with endogenous GARP/EARP. HEK293FT cells were either mock transfected or transfected with GUS or with FLAG-RAB29. Protein extracts were subjected to IP with anti-FLAG antibodies and immunoblotted for endogenous LRRK2, VPS52, VPS52, and VPS53 and FLAG-protein baits. (G) EARP/GARP components interact with LRRK2. HEK293FT cells were transfected with GFP-tagged versions of VPS50 (syndetin) and VPS54. Both proteins could interact with FLAG-tagged LRRK2. (H) Knockdown (KD) of VPS52 leads to decreased LRRK2 and Rab29 expression. HEK293FT cells were subject to siRNA KD of either NTC or VPS52 and then probed for endogenous LRRK2, VPS52, RAB29, and β-actin. (I and J) Quantification of endogenous LRRK2 and RAB29 following NTC or VPS52 siRNA treatment of HEK293FT cells. There is a significant decrease in endogenous LRRK2 and RAB29 following KD of VPS52 following normalization to endogenous β-actin and using t test. Error bars represent SEM between replicates for each group.

To validate the physical interactions predicted by our extended protein arrays, we immunoprecipitated tagged RAB29 and recovered endogenous VPS52 and endogenous LRRK2 (Figure 2A). Reciprocally, pull-down of tagged VPS52 indicated interaction with endogenous LRRK2 (Figure 2B). Finally, immunoprecipitation (IP) of tagged LRRK2 recovered endogenous VPS52 (Figure 2C), indicating that all three proteins can interact in cells.

VPS52 has been shown to be a subunit of two complexes, GARP and EARP, which are important in retrograde protein sorting to the TGN and recycling from RAB4-positive endosomes respectively (Bonifacino and Hierro, 2011; Pérez-Victoria and Bonifacino, 2009; Pérez-Victoria et al., 2008). Extending the subnetwork of protein interactions here with publicly available data identified that VPS53 and VPS51 would also be part of the GARP complex (Figure 2D). IPs using either LRRK2 (Figure 2E) or RAB29 (Figure 2F) contained VPS52 as well as VPS53 and VPS51, demonstrating that LRRK2 and RAB29 bind the intact GARP or EARP complexes not simply free VPS52. Additionally, we found that LRRK2 co-immunoprecipitated with both VPS50/syndetin and VPS54, showing that LRRK2 could interact with either GARP or EARP (Figure 2G).

Knockdown of VPS52 resulted in decreases in LRRK2 and RAB29 levels (Figures 2H, 2I, and 4B; Figure S5B), as has been shown before for other subunits of GARP complex for which depletion of one destabilizes the others (Pérez-Victoria et al., 2008).

Next, we performed mapping experiments to identify the domain(s) of LRRK2 that are responsible for interaction with VPS52. Interestingly, only full-length LRRK2 was able to strongly co-immunoprecipitate endogenous VPS52 in cells (Figure S2A), suggesting that multiple domains of LRRK2 are required to bind VPS52. We also examined interactions between domains of VPS52 and LRRK2. We confirmed prior data that the N-terminal region of VPS52 was required for interaction with other components of the GARP complex but found, in contrast, that deletion constructs exhibited a weakened interaction with LRRK2, suggesting that both N- and C-terminal regions of VPS52 are important for binding to LRRK2 (Figure S2B). Interestingly, the same deletion constructs showed a diffuse cytoplasmic localization in cells, while only full-length VPS52 was localized to organellar structures in cells. This demonstrates that the correct localization of VPS52 depends on both C- and N-terminal domains, similar to the binding to LRRK2.

### LRRK2 and RAB29 Influence VPS52 Dynamics

**Figure 3.**
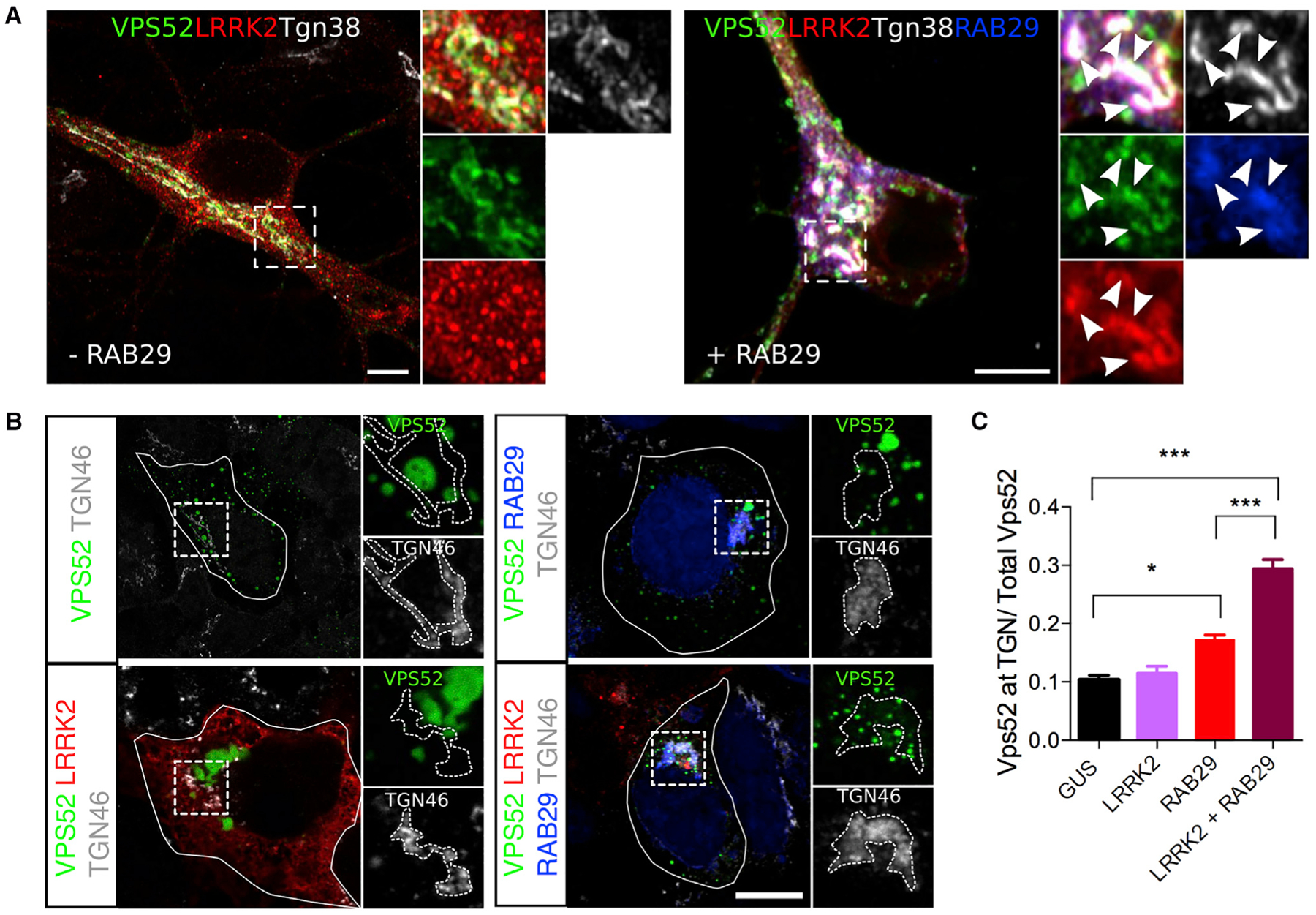
LRRK2:RAB29 Affect VPS52 Dynamics (A) Airyscan pictures showing LRRK2 complex components co-localize at the TGN. Transfection of tagged versions of VPS52 (green), RAB29 (blue), and LRRK2 (red) in primary mouse neurons demonstrates co-localization at the TGN (gray) only in the presence of RAB29 (right panel). White arrowheads indicate co-localization. Scale bar: 5 μM. (B) RAB29 and LRRK2 promote VPS52 recruitment to the TGN. Cells were transfected with VPS52 (green) either alone or with LRRK2 (red), RAB29 (blue), or both and stained with the TGN marker TGN46. Scale bar: 10 μM. (C) Quantification of the relative proportion of VPS52 in the region of the TGN relative to all VPS52 per cell. There was a statistically significant difference in the proportion of VPS52 staining at TGN relative to overall staining across the cell between groups (one-way ANOVA, N = 3 experiments). Individual comparisons were made using Tukey’s post hoc test; data are represented as mean ± SEM.

We next attempted to understand the relationship among LRRK2, VPS52, and trafficking in cells. First we established the cellular patterns of LRRK2 and VPS52 expression. In the mouse brain, the mRNAs for both Lrrk2 and Vps52 were expressed broadly across brain regions in a similar pattern (Figure S3A). On the basis of our initial screening experiments (Figures 1C and 1D), we predicted that LRRK2 and VPS52 would co-localize to TGN or vesicular structures containing GARP or EARP, respectively. Consistent with this hypothesis, using fixed cells, we found that GFP-tagged VPS52 was present at the TGN (as VPS52 co-localized with the mouse TGN marker Tgn38) in primary neurons (Figure S3B). LRRK2 was found to co-localize with VPS52 at the TGN, especially when RAB29 was present (Figure 3A).

Similarly, LRRK2 co-localized with VPS52 in transfected HEK293FT cells, and addition of RAB29 promoted relocalization of the two proteins to the perinuclear area (Figure S4A). These results were specific for VPS52, as we did not find co-staining of LRRK2 and VPS35 (Figure S4A), a retromer component that can be mutated in some cases of inherited PD (Zimprich et al., 2011). To provide a more quantitative assessment of the roles of GARP/EARP in recruitment of LRRK2 to the TGN, we used the same high-content imaging approach previously used to validate VPS52 and also included additional VPS proteins. We found that treatment with siRNA against VPS52 decreased LRRK2:RAB29 localization to the TGN, but knockdown of VPS35 or VPS29 (another retromer component) had no effect, showing that any interactions between LRRK2, RAB29, and VPS52 are independent of retromer (Figure S4B). Knockdown of VPS52 or the GARP components VPS53 and VPS51 also decreased localization of LRRK2 to the TGN but knockdown of VPS54 or syndetin/VPS50 did not (Figure S4B). These results collectively show that RAB29 recruits LRRK2, that VPS52 supports this recruitment to the TGN, and that GARP plays a specific role in LRRK2:RAB29 interactions at the TGN.

We next sought to assess the possible effect of LRRK2:RAB29 on VPS52 localization and dynamics. We observed that RAB29 increased the VPS52 staining at the TGN relative to overall levels, and this was further enhanced by LRRK2 (Figures 3B and 3C). These observations indicate that LRRK2 and RAB29 work together to influence localization of VPS52-containing structures.

### LRRK2:RAB29 Stabilize the SNARE-GARP Complex Interaction at the TGN

**Figure 4.**
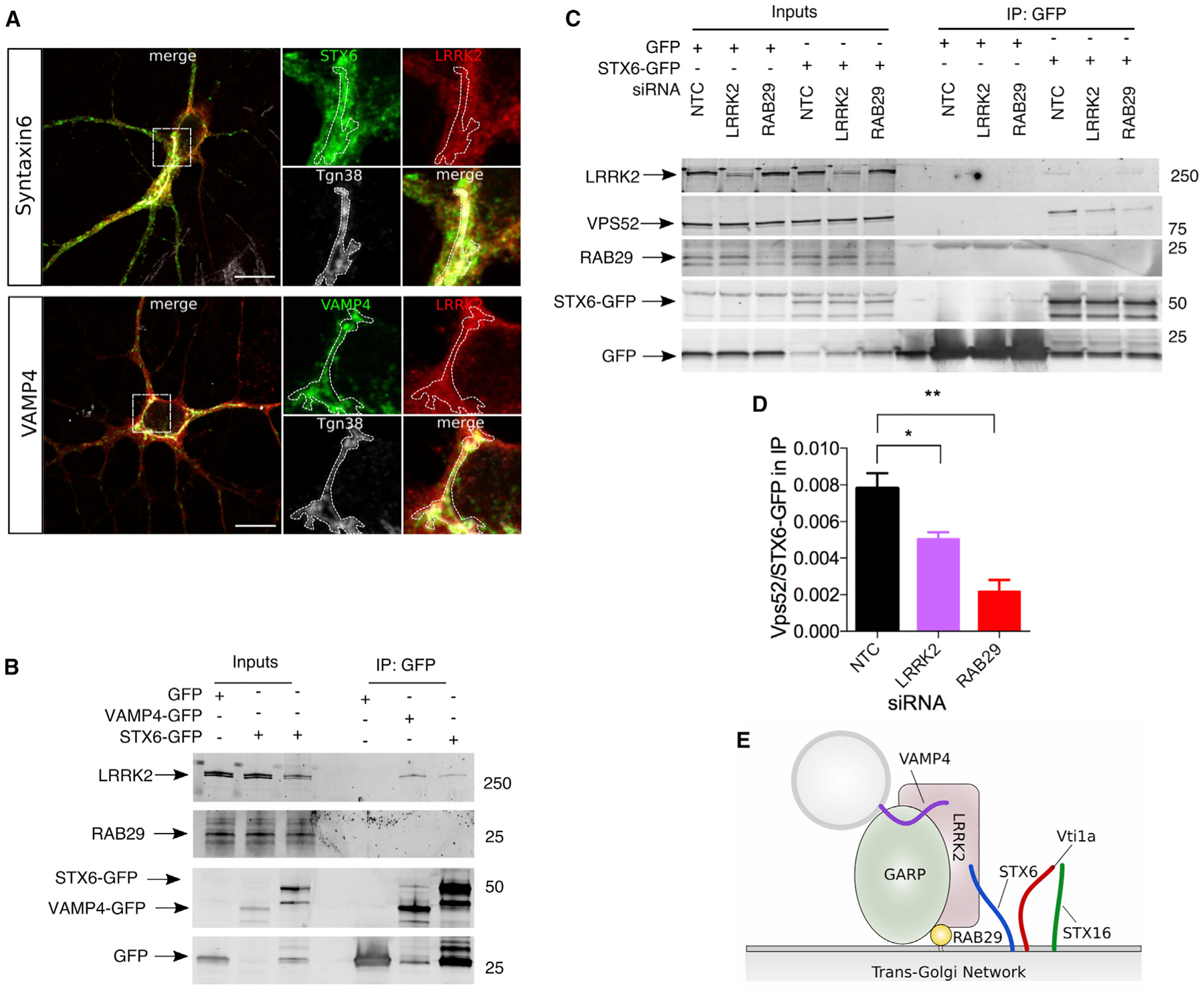
LRRK2 Stabilizes Interactions between GARP and SNARE Proteins at the TGN (A) Syntaxin-6 and VAMP4 localization in neurons. Images of primary mouse cortical neurons expressing Syntaxin-6 (upper panels, green) or VAMP4 (lower panels, green) and LRRK2 (red) and stained for the mouse TGN marker Tgn38 (gray). Both SNARE proteins are found at the TGN in these cells. Scale bar: 10 μM. (B) Syntaxin-6 and VAMP4 interact with endogenous LRRK2. GFP-tagged versions of VAMP4 and Syntaxin-6, or GFP alone, were transfected into HEK293FT cells. Both proteins could co-immunoprecipitate endogenous LRRK2 but did not appear to interact with RAB29. (C) LRRK2 and RAB29 stabilize GARP-SNARE interactions. GFP-tagged STX-6 or GFP alone was transfected into HEK293FT cells that were also treated with siRNA to KD endogenous LRRK2 or RAB29, with a NTC siRNA as a negative control for KD. (D) Quantification of interactions between VPS52 and Syntaxin-6. Relative interaction between GFP-tagged Syntaxin-6 and endogenous VPS52 as in (C) was quantified across n = 3 replicates. There was a significant effect of siRNA treatment group by one-way ANOVA compared with NTC siRNA by Tukey’s post hoc tests. Data are represented as mean ± SEM. (E) A working model of the interactions among LRRK2, RAB29, GARP, and SNARE proteins at the TGN.

To explore the mechanism by which LRRK2 and RAB29 co-operate with the GARP complex, we next examined which proteins at the TGN mediate fusion events. We confirmed, in primary neuron cultures, prior data showing that the t-SNARE Syntaxin-6 (STX-6) (Bock et al., 1997) and the v-SNARE VAMP4 (Mallard et al., 2002) are partially localized to the TGN (Figure 4A). Both SNAREs play an integral part in endosome-TGN retrograde transport, and the assembly of the v-SNARE/t-SNARE complex is GARP complex dependent (Mallard et al., 2002; Pérez-Victoria and Bonifacino, 2009). Co-IP showed that pull-down of STX-6 or VAMP4 was able to recover LRRK2, but under the same conditions, RAB29 was not detected in co-IP (Figure 4B). These results show that LRRK2 interacts independently with both RAB29 and the v-SNARE/t-SNARE complex.

We next examined whether LRRK2:RAB29 might play a role in interactions between GARP and SNARE proteins. Using co-IP, we were able to confirm the previously reported (Pérez-Victoria et al., 2010) interaction between STX-6 and endogenous VPS52 (Figure 4C). We additionally found that knockdown of endogenous LRRK2 or RAB29 diminished interaction between VPS52 and STX-6 (Figures 4C and 4D). Collectively, these data support a model in which LRRK2 strengthens GARP/SNARE interactions at the TGN by simultaneously binding both protein complexes (shown schematically in Figure 4E).

### LRRK2 and RAB29 Promote Efficient Vesicle Trafficking

Having validated the physical interactions between LRRK2, RAB29 and GARP and demonstrated that LRRK2 and RAB29 preserve the GARP-SNARE complex formation, we next asked whether these events have effects on vesicular trafficking in the cell. For these experiments, we focused on the contribution of endogenous components of the protein complex to trafficking of cargoes within cells (Pfeffer, 2009), using primary mouse astrocytes, as these cells highly express endogenous Lrrk2 and are suitable for trafficking assays because of their large surface area in culture.

**Figure 5.**
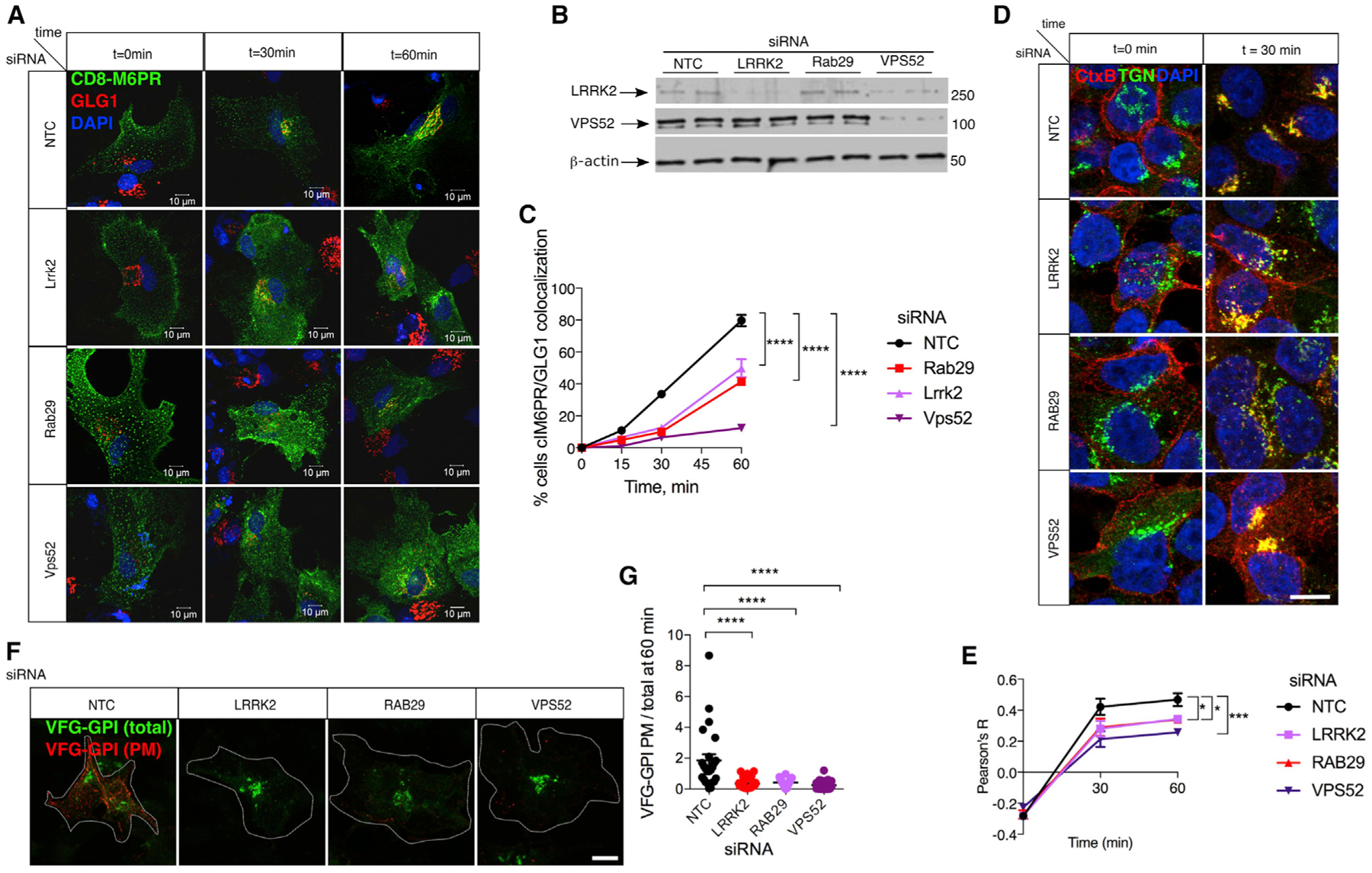
LRRK2 Promotes Bidirectional Transport to and from the TGN (A) Imaging of retrograde transport from the plasma membrane to the TGN. Trafficking of CD8 tagged CI-M6PR (green) from the plasma membrane to the TGN (marked with GLG1, red) was followed at the indicated times after shift from 4°C to 37°C in primary mouse astrocytes treated with a NTC or siRNA against Lrrk2, RAB29, or Vps52. N = 3 independent experiments. Scale bar: 10 μM. (B) Parallel duplicate samples from the experiment in (A) were blotted for (from top to bottom) Lrrk2, Vps52, and β-actin as a loading control. Lrrk2 levels fall in parallel to Vps52. N = 3 independent experiments. (C) Quantification of retrograde trafficking from the plasma membrane to the TGN. The numbers of cells for which CD8-M6PR had been retrogradely trafficked to the TGN over time after treatment with a NTC (black line), Lrrk2 (light purple), Rab29 (red), or Vps52 (violet) siRNA. Data are mean ± SD from N = 3 independent experiments with at least 100 cells counted in each experiment. Two-way ANOVA showed a significant effect of both time and siRNA. Multiple comparisons are shown for the siRNAs using Tukey’s post hoc test. (D) HEK293FT cells were treated with indicated siRNA against LRRK2, RAB29, or VPS52 (NTC). CtxB (red) was added at time 0 (left panels) and chased at 37°C for 30 min (right panels), then stained for TGN (green). Scale bar: 10 μM. (E) Quantification of co-localization between CtxB and TGN over time after treatment with siRNAs against LRRK2 (light purple), RAB29 (red), or VPS52 (violet). Using two-way ANOVA, there was a significant effect of time and of siRNA. Individual siRNAs were compared back to NTC using Dunnett’s post hoc test; N = 3 experiments. Data are represented as mean ± SEM. (F) Imaging of post-Golgi anterograde trafficking. Cells were treated with NTC or siRNA against LRRK2, RAB29, or VPS52 and transfected with a VSVG-FLAG-GFP-GPI (VFG-GPI) vector. To detect VFG-GPI at the plasma membrane, cells were stained with FLAG-M2 antibody (red) in non-permeabilized conditions and compared with GFP intracellular VFG-GPI (green). Scale bar: 10 μM. (G) Quantification of anterograde trafficking. The amount of VFG-GPI at the plasma membrane relative to total VFG -GPI was calculated on a per cell basis across three independent experiments. Outliers were removed using ROUT with Q = 1%, which removed two data points from the NTC, four from LRRK2, and none from the other two siRNA groups. There was a significant effect of siRNA treatments by one-way ANOVA. Effects of individual siRNAs were analyzed using Dunnett’s post hoc test, compared with NTC, n = 23–35 cells. Data are represented as mean ± SEM. N = 3 independent experiments.

To estimate effects of LRRK2 complexes on retrograde trafficking, we used cell surface labeling of CD8-M6PR fusion proteins on living primary astrocytes and followed, over time, the internalization of antibodies against CD8 (McKenzie et al., 2012; Wang et al., 2014). CD8-M6PR was trafficked to the TGN in ~74% of control cells after 1 h of chase. In contrast, internalization was markedly diminished in astrocytes in which endogenous Vps52, Rab29, or Lrrk2 was knocked down using siRNA (Figures 5A–5C). Similar results were seen with endogenous LRRK2, RAB29, and VPS52 in HEK293FT cells (Figures S5A, S5B, and S5E). To make sure the M6PR trafficking defects were not a consequence of an impairment of plasma membrane to early endosome trafficking, we analyzed the co-localization of CD8-M6PR with early endosomal marker EEA1 after a 15 min chase. We found that 80%–90% of cells internalized CD8-M6PR, and we did not find statistically significant differences in internalization between groups (Figures S5C and S5D). We also noted, as reported in Figures 2H and 2I, a destabilization of LRRK2 when VPS52 was knocked down in both primary astrocytes (Figure 5E) and in HEK293FT cells (Figure S4B), consistent with complex formation between these proteins.

The effects of LRRK2 and interacting proteins on retrograde transport of CI-M6PR led us to ask if this pathway was specifically affected by LRRK2 deficiency or whether there was a more generalized effect on trafficking. To distinguish these possibilities, we used the well-characterized retrograde trafficking of cholera toxin B (CtxB) from the cell surface to the TGN, a pathway that depends on binding of cholera toxin to the ganglioside GM1 rather than CI-M6PR. We found that knockdown of LRRK2, RAB29, or VPS52 would each diminish the efficiency of retrograde trafficking of CtxB to the TGN (Figures 5D and 5E). This was particularly severe in cells lacking VPS52, in which CtxB trafficking to the TGN was reduced by ~50%, in agreement with previous reports on the effect of GARP or STX-6 in CtxB retrograde transport (Ganley et al., 2008; Hirata et al., 2015). These results demonstrate that LRRK2 and associated proteins have a generalized effect on retrograde transport of multiple cargoes.

Previous data have suggested that disruption of GARP function can also affect anterograde transport, as recycling of proteins by GARP to the TGN is required for efficient post-Golgi transport of GPI proteins (Hirata et al., 2015). Given that LRRK2, RAB29, and VPS52 promoted retrograde transport of different cargoes to the TGN via GARP, we hypothesized this complex might also affect anterograde transport in cells. To test this hypothesis, we measured the anterograde transport of GPI proteins from the TGN to the plasma membrane using the VFG-GPI construct (Hirata et al., 2015). Consistent with our predictions, knockdown of endogenous LRRK2, RAB29, or VPS52 also diminished anterograde transport 1 h after chase at 32°C (Figures 5F and 5G).

**Figure 6.**
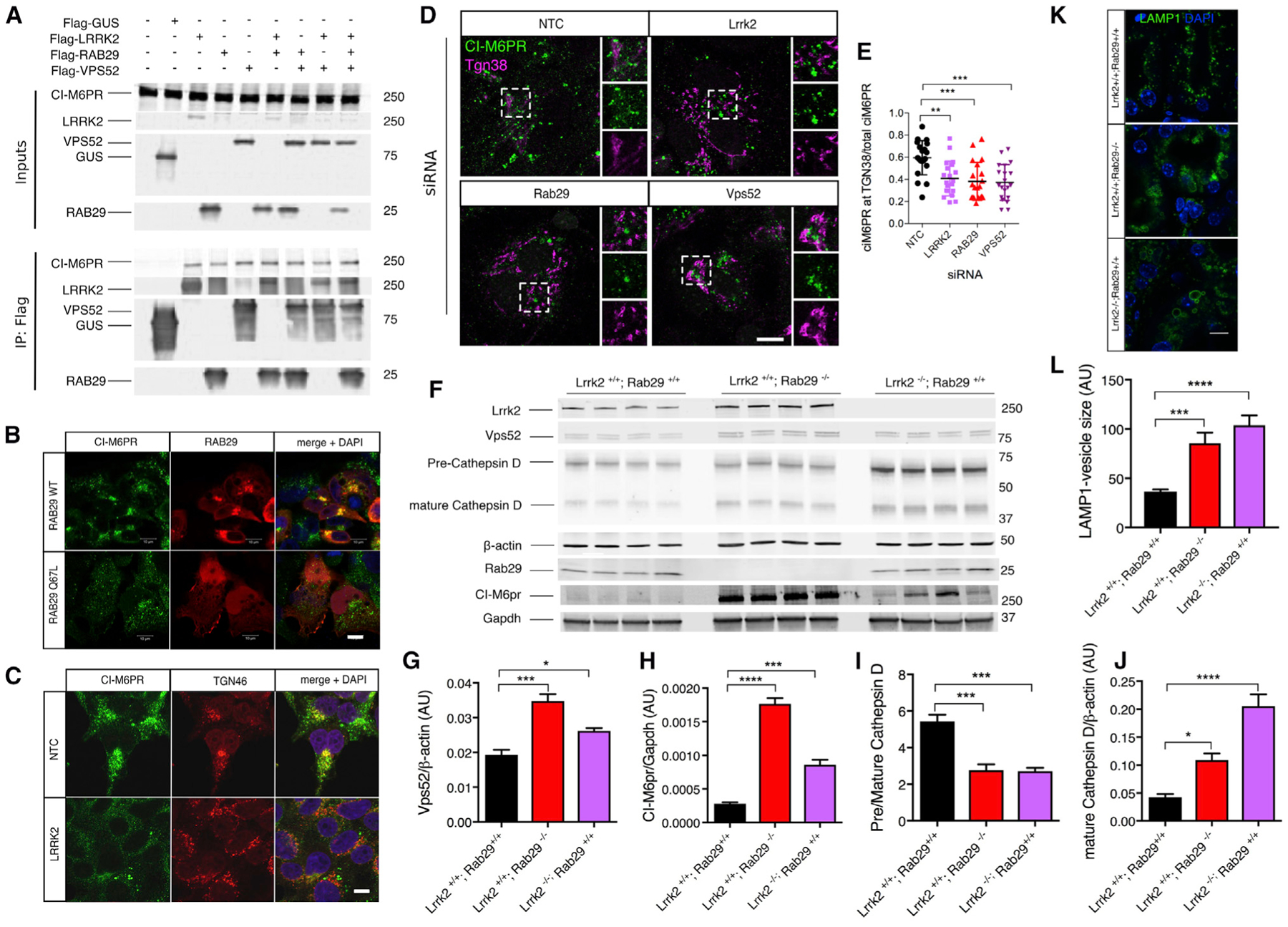
LRRK2 and Interacting Partners Regulate CI-M6PR Trafficking and Processing of Cathepsin D (A) LRRK2, VPS52, and Rab29 interact with endogenous CI-M6PR. HEK293FT cells were transfected with FLAG-tagged versions of LRRK2, RAB29, and VPS52. Protein lysates were subject to IP for FLAG and blotted for endogenous CI-M6PR or FLAG proteins. CI-M6PR is recovered in all IP materials. (B) HEK293FT cells were transfected with WT or Q67L RAB29 constructs (red) and stained for CI-M6PR (green). Merged images on the right include DAPI for nuclei. Scale bar: 10 μM. (C) CI-M6PR partially co-localizes to the TGN in a LRRK2-dependent manner. HEK293FT cells were stained for endogenous CI-M6PR (green) and TGN46 (red) after treatment with NTC siRNA or siRNA against endogenous LRRK2 (lower panels). Scale bar: 10 μM. (D) Imaging of endogenous CI-M6PR at the TGN. Primary astrocytes were stained for endogenous CI-M6PR (green) and Tgn38 (magenta) after treatment with NTC siRNA or siRNA against endogenous Lrrk2, Rab29, or Vps52. Scale bar: 10 μM. (E) Quantification of cells as in (D) for the amount of CI-M6PR at the TGN. For each cell, we quantified the relative amount of CI-M6PR staining overlapping with Tgn38 compared with all CI-M6PR in the cell. One-way ANOVA with Tukey’s post hoc test was used; n = 20 cells. Data are represented as mean ± SD. (F) Kidneys from 9-month-old WT, Rab29-KO or Lrrk2-KO mice were extracted and blotted for endogenous CI-M6pr, Lrrk2, Vps52, cathepsin D, Rab29, or the loading control β-actin and Gapdh. (G) Quantification of Vps52 protein levels in mouse kidneys. One-way ANOVA (N = 4 animals per genotype). Individual comparisons are indicated above each bar; with Dunnett’s post hoc test compared with WT (Lrrk2^+/+^; Rab29^+/+^) animals. Data are represented as mean ± SEM. (H) Quantification of endogenous mature cathepsin D in WT or KO animals relative to β-actin. One-way ANOVA (N = 4). Individual comparisons are indicated above each bar, by Dunnett’s post hoc test compared with WT animals. Data are represented as mean ± SEM. (I) Quantification of endogenous pre-cathepsin D in WT or KO animals relative to mature cathepsin D. One-way ANOVA (N = 4). Individual comparisons are indicated above each bar, with Dunnett’s post hoc test compared to WT animals. Data are represented as mean ± SEM. (J) Quantification of endogenous CI-M6pr in WT or KO animals relative to Gapdh. One-way ANOVA (N = 4). Individual comparisons are indicated above each bar, with Dunnett’s post hoc test compared to WT animals. Data are represented as mean ± SEM. (K) Histological characterization of Lrrk2- and Rab29-KO kidneys for Lamp1 and DAPI staining. Scale bar: 10 μm. (L) Area quantification of LAMP1-positive structures in all three groups. One-way ANOVA. Individual comparisons are indicated above each bar, with Dunnett’s post hoc test compared to WT animals. Data are represented as mean ± SEM.

We next asked what the effect of LRRK2 and interacting partners was on trafficking of endogenous cargo proteins in cells, in addition to the reporter molecules used above. First, we noted that LRRK2 and its interacting partners can bind the cargo protein CI-M6PR. Pull-down of tagged LRRK2, RAB29, and VPS52 recovered endogenous CI-M6PR (Figure 6A). Next, we noted that expression of RAB29 promoted recruitment of endogenous CI-M6PR to perinuclear structures, but this was not seen with Q67L-RAB29, which we have previously shown does not retain GTP/GDP (Beilina et al., 2014; Figure 6B). Conversely, knockdown of LRRK2 decreased co-localization of CI-M6PR with TGN46 (Figure 6C). These results demonstrate that localization of CI-M6PR to the TGN depends on both LRRK2 and active RAB29. To further demonstrate that the LRRK2 complex is involved in recycling of cargo proteins, we additionally examined localization of endogenous CI-M6PR with the TGN in primary astrocytes. Knockdown of each of the complex components, Lrrk2, Rab29, and Vps52, resulted in a decrease of CI-M6PR levels in the TGN compared with the overall cellular levels (Figures 6D and 6E).

CI-M6PR is critical for correct trafficking of lysosomal enzymes, which allowed us to examine if there were correlates of altered trafficking *in vivo*. Despite showing no strong neurological phenotype, there are reproducible age-dependent changes in markers of vesicular transport and autophagy in the kidney of Lrrk2 knockout (KO) animals (Herzig et al., 2011; Tong et al., 2010, 2012). We therefore examined mice lacking either Lrrk2 or Rab29 and found an accumulation of precursor and mature cathepsin D in both Lrrk2- and Rab29-KO mice (Figures 6F, 6H, and 6I; in agreement with previous reports claiming lysosomal dysregulation in the kidney of Lrrk2-KO mice; Kuwahara et al., 2016; Pellegrini et al., 2018; Tong et al., 2012), as well as an increase in the levels of Ci-M6pr (Figures 6F and 6J). Moreover, Vps52 protein levels were significantly higher in both genotypes of KO mice compared with wild-type (WT) littermate controls (Figures 6F and 6G). Additionally, by staining for the late endosomal/lysosomal marker Lamp1 in Lrrk2- and Rab29-KO animals, we observed a strong increase in the size of Lamp1-positive structures compared with WT kidneys (Figures 6K and 6L), consistent with lysosomal dysregulation. Although we cannot rule out that some of these events are due to compensatory changes to the loss of Lrrk2/Rab29 *in vivo*, it is also likely that they arise from prolonged mis-sorting of cargo and generalized defects in trafficking. In either case, these results show that endogenous Lrrk2 and interacting proteins promote the efficient cycling of CI-M6PR at the TGN, which in turn controls the homeostasis of lysosomal enzymes such as cathepsin D, including *in vivo*.

### Pathogenic LRRK2 Mutations Associated with PD Deregulate Bi-directional Trafficking and Are More Sensitive to Ceramide-Induced Toxicity

The collective observations outlined above advance a model in which loss of LRRK2 and its interacting partners RAB29 and VPS52 leads to less efficient retrograde transport to the TGN, resulting in trafficking defects and mis-sorting of lysosomal enzymes. To understand what properties of LRRK2 might be involved in these processes, we repeated these experiments with mutations that would either increase or decrease known enzymatic functions of the protein. Compared with WT protein, both R1141C and G2019S increased the size of large VPS52-positive structures while T1348N and K1906M decreased the number of these structures (Figure S6A).

**Figure 7.**
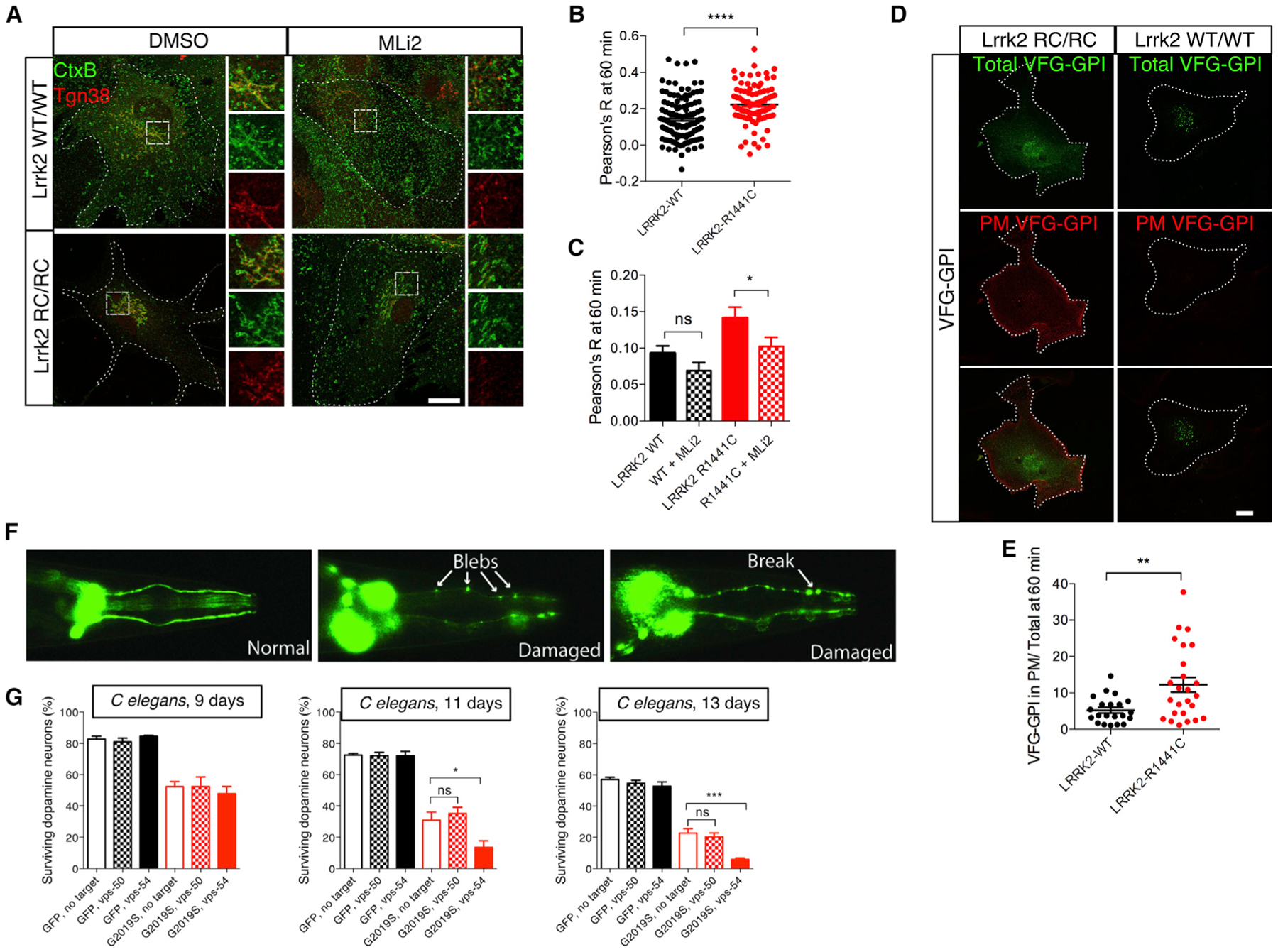
Pathogenic LRRK2 Induces Trafficking Defects and Interacts Genetically with GARP to Promote Toxicity *In Vivo* (A) Endogenous mutation in Lrrk2 promotes retrograde trafficking from the plasma membrane to the TGN. Primary astrocytes from WT or Lrrk2 R1441C-knockin animals were used for CtxB (green) chase to the TGN (Tgn38 staining, red) for 60 min at 37°C. Scale bar: 20 μm. (B) Replication of retrograde trafficking effect of R1441C Lrrk2 in an additional experiment with n > 100 cells per genotype. Statistical comparison between genotypes was made using two-tailed Student’s t test. Data are mean ± SEM. N = 3 independent experiments. (C) Quantification of retrograde trafficking. The correlation between CtxB and Tgn38 (Pearson’s R) for n > 72 cells across three experiments from WT or R1441C-knockin astrocytes. Statistical comparisons were made between untreated and MLi-2-treated samples using Student’s t test. Data are mean ± SEM. N = 3 independent experiments. (D) Endogenous mutation in Lrrk2 promotes anterograde trafficking from TGN to plasma membrane. Primary astrocytes from WT or R1441C-knockin animals were transfected with VFG-GPI (green) and cell surface VFG-GPI (red) stained using fixed, non-permeabilized conditions. Scale bar: 10 μm. (E) Quantification of anterograde trafficking. Graphs show the amount of VFG-GPI relative to total across cells after 60 min incubation at 37°C. Statistical comparison between genotypes was made using two-tailed Student’s t test after removal of three outlier values per genotype using ROUT (Q = 1%), n = 24–28 cells. Data are mean ± SEM. N = 3 independent experiments. (F) Imaging dopamine neuron degeneration in *C. elegans*. Representative images are shown of healthy (left) and degenerating dopamine neurons (right two panels) in the G2019S *C. elegans* model. (G) Quantification of toxicity by LRRK2 and GARP or EARP KD. *C. elegans* expressing either GFP (green bars) or G2019S-LRRK2 (red bars) were treated with RNAi against VPS54 or VPS50 as indicated. Dopamine neurons were counted at 9, 11, and 13 days of adult lifespan. Bonferroni post hoc test comparing siRNA for VPS54 or VPS50 to NTC from one-way ANOVA. Bars show the mean number of surviving dopamine (±SEM) neurons across cohorts of 100 worms from N = 4 independent experiments.

To address whether the same effects might be seen at the endogenous level, we took advantage of mice with the pathogenic R1441C (Tong et al., 2009) mutation knocked in to the endogenous mouse Lrrk2 locus. Using this model, we found that CtxB translocation to the TGN was markedly higher in primary astrocytes from R1441C knockin animals compared with their WT littermates (Figures 7A and 7B). Furthermore, the LRRK2 kinase inhibitor MLi2, which we confirmed causes dephosphorylation of LRRK2 and prevents RAB29-dependent recruitment to the TGN (Figures S6B–S6D), could reverse the effects of the R1441C mutation on CtxB trafficking (Figures 7A and 7C). Anterograde trafficking was also affected by the endogenous R1441C mutation in primary astrocytes, as shown by enhanced trafficking of VSVG-GPI to the cell surface (Figures 7D and 7E). Together, these results show that mutations in LRRK2 are gain of function for effects on VPS52 trafficking.

### GARP Depletion Exacerbates LRRK2 Toxicity *In Vivo*

Collectively, the above results demonstrate that pathogenic LRRK2 enhances trafficking and that in a mammalian system interacts with RAB29 and VPS52 to positively affect retrograde transport to the TGN. To address whether LRRK2 and GARP or EARP contribute to pathophysiological events, we took an *in vivo* approach. We used an established model of neuronal toxicity in which human G2019S LRRK2 is expressed in the dopaminergic neurons of *C. elegans.* As previously reported, this model has age-dependent loss of dopamine neurons (Figures 7F and 7G). Interestingly, toxicity in this model was exacerbated by RNAi against the GARP subunit vps-54 but not by the EARP subunit vps-50 (Figure 7G). These results suggest that toxicity of mutant LRRK2 in dopamine neurons is ameliorated by functional GARP.

## DISCUSSION

Although categorization of the function of all PD risk genes is incomplete, variation in genes encoding endo-lysosomal proteins appears to play a strong role in PD risk (Bras et al., 2008; Sidransky et al., 2009). Indeed, identifying the normal function of LRRK2 has important implications both for understanding etiopathogenesis of PD and for whether kinase inhibition will be a useful strategy to treat this disease. Here, by identifying and validating interaction partners of LRRK2 that include the GARP complex, we uncover a function of LRRK2 in intracellular protein recycling and describe mechanisms by which LRRK2 could influence localization of lysosomal enzymes. These results also link LRRK2 to several known PD genes involved in endosomal membrane trafficking.

Several groups have now confirmed that LRRK2 interacts with RAB29 (Beilina et al., 2014; Kuwahara et al., 2016; Liu et al., 2018; MacLeod et al., 2013; Purlyte et al., 2018), demonstrating that the protein array approach we originally used to identify this pair can be used to discover reliable protein interactions. We extended this approach to nominate a network of candidate interactors for LRRK2, RAB29, GAK, and BAG5.

Prior results demonstrated that RAB29 is present at the TGN (Beilina et al., 2014; Kuwahara et al., 2016; Liu et al., 2018; MacLeod et al., 2013; Purlyte et al., 2018). We therefore determined which, if any, of the identified candidate interactions of LRRK2 were important for LRRK2 to be recruited to the TGN by RAB29. The identification of interaction of LRRK2 with VPS52, and by extension the GARP complex, has important functional implications for the cell. Working through some of the effects on cells allowed us to show that LRRK2 and GARP influence both anterograde and retrograde trafficking, mechanistically by increasing the interaction of VPS52 with the SNARE protein STX-6. We infer that this interaction results in a greater efficiency of heterotypic membrane fusion. This concept is consistent with our *in vivo* observations that mice lacking Lrrk2 and Rab29 upregulate Vps52 to likely compensate for lack of functional regulation.

Using these measures of LRRK2 function, we have shown that mutations in LRRK2 promote trafficking. This is consistent with the known biochemical effect of mutations such as R1441C that lower GTPase activity (Daniëls et al., 2011; Lewis et al., 2007; Li et al., 2007; Liao et al., 2014), thus leaving LRRK2 in a GTP-bound state. It is important to consider models in which efficient trafficking and recycling of components such as CI-M6PR might lead to cellular dysregulation relevant to PD. Indeed, in agreement with our observations, postmortem analysis of the frontal cortex from PD patients harboring LRRK2 mutations in the kinase domain (G2019S and I2020T) displayed a marked reduction in CI-M6PR protein expression (Zhao et al., 2018). This strongly suggests that CI-M6PR mis-sorting is an important event during LRRK2-related PD. Our *in vivo* data show that M6PR dysregulation is accompanied by an alteration in the levels of its cargo, cathepsin D, implying lysosomal distress. It is worth noting that we observed enlarged lysosomes in the kidneys of our KO mice, which could be the result of non-degraded cargo due to impaired trafficking. This observation is in line with the known effect of GARP disruption in lysosomal morphology (Gershlick et al., 2019; Pérez-Victoria et al., 2008). Dysregulation of lysosomal proteins in LRRK2 PD cases is not just limited to cathepsin D, as significant decreases in GBA levels have also been reported in postmortem brains of LRRK2 mutant cases (Zhao et al., 2018). Furthermore, prior published work from our group has shown that like cathepsin D, the expression of Legumain, another lysosomal enzyme, is also altered in the kidneys of LRRK2 KO mice (Pellegrini et al., 2018). Support for the notion that mistrafficking of lysosomal proteins is a trigger for PD also comes from large-scale genetic studies in which a number of nominated genes demonstrated a connection to the endolyso-some system. Specifically *GBA1*, *SCARB2*, *TMEM175*, *CTSB*, *GALC*, and *ATP6V0A1* were found in the last two GWASs (Chang et al., 2017; Nalls et al., 2018) to be associated with an increase in the risk for developing PD, while *ATP13A2* is linked to familial PD. Furthermore, Robak et al. (2017) demonstrated that lysosomal storage disease variants increase the risk for PD and further nominated *CTSD* (the gene that encodes for cathepsin D) as a candidate PD susceptibility gene. Pathologically PD is characterized by insoluble and aggregated α-synuclein, packed into intracellular inclusions termed Lewy bodies. Animal and cellular studies have demonstrated that CTSD is one of the main lysosomal endopeptidases responsible for the degradation of α-synuclein (Cullen et al., 2009; Sevlever et al., 2008), and marked increases in aggregated levels of α-synuclein have been observed in CTSD-KO mice (Qiao et al., 2008). Thus, as α-synuclein is predominantly degraded through lysosomal pathways (Cuervo et al., 2004; Gao et al., 2008), we speculate that chronic lysosomal dysfunction may be important in the formation of PD Lewy pathology.

We have not yet confirmed that the effects of manipulation of GARP, and not EARP, on mutant LRRK2 toxicity in *C. elegans* can be extrapolated to mammalian species or to humans. However, with regard to PD, the importance of the TGN retrograde transport, in which GARP plays a role, is highlighted by the implication of several genes associated with the pathway to disease pathogenesis. *VPS35*, *DNAJC13*, and *PLA2G6* mutations are responsible for familiar cases, and their proteins regulate the sorting of cargo from the endosomes to the TGN by controlling retromer function (Lin et al., 2018; McGough et al., 2014; Shi et al., 2009). Additionally, we demonstrate that LRRK2 physically interacts with the v-SNARE VAMP4, and interestingly, the latest GWAS identifies *VAMP4* as a risk factor for PD (Nalls et al., 2018).

The present data would indicate that loss of GARP function would not be a valid strategy for PD therapeutics, but models in which gain of LRRK2 function is associated with cellular dysfunction support the concept that kinase inhibition might be therapeutically useful in PD patients (West, 2015). We show here that the potent LRRK2 inhibitor MLi-2 can reverse mutant LRRK2-induced trafficking defects in cells, consistent with the beneficial effects of LRRK2 inhibitors in a number of models of neurodegeneration relevant for PD (Daher et al., 2015; Volpicelli-Daley et al., 2016; Yao et al., 2013). Our results therefore identify an underlying cellular pathway that represents a valid therapeutic target for PD, opening up possibilities for combination therapy against both LRRK2 and lysosomal disease mechanisms.

## STAR★METHODS

### RESOURCE AVAILABILITY

#### Lead Contact

Further information and requests for resources and reagents should be directed to and will be fulfilled by the Lead Contact, Mark R. Cookson (cookson@mail.nih.gov).

#### Materials Availability

Plasmids not covered by any restrictions like MTAs generated in this study are available upon request. Rab29 knockout mouse line generated in this study will be available upon request.

#### Data and Code Availability

The published article includes Protein array and TGN screen datasets generated and analyzed during this study (Tables S1 and S2).

### EXPERIMENTAL MODEL AND SUBJECT DETAILS

#### Mice

All procedures with animals followed the guidelines approved by the Institutional Animal Care and Use Committee of National Institute on Aging. Experiments were performed on adult C57BL/6: wild-type, LRRK2 knockout, and Rab29 knockout mice. We used mice of both sexes in this study. We used 9-month-old and P0 pups in this study.

#### Cell lines, primary culture

HEK293FT cells were maintained in DMEM containing 4.5 g/l glucose, 2 mM l-glutamine, and 10% fetal bovine serum at 37°C in 5% CO_2_. Primary cortical and astrocytes culture were made from C57BL/6 newborn pups. Primary cortical neurons were cultured in BME supplemented with B27, N2, 1 mM glutaMAX, 0.45% glucose, and 2.5 μM cytosine arabinoside. Primary astrocytes were cultured in DMEM media containing 10% FBS. HEK293FT cells and primary neuronal cultures were seeded on 12 mm coverslips pre-coated with poly-D-lysine (Corning).

#### C.elegans strains growth and propagation

All strains were maintained on NGM agar seeded with OP50 using standard methods. The GFP and G2019S LRRK2 lines which express GFP or GFP/G2019S LRRK2 in dopaminergic neurons were generously provided by Dr. Shu Chen (Yao et al., 2010). The TU3401 line with neuronal expression of sid-1 in a sid-1 null background (hypersensitive to neuronal RNAi), was obtained from the Caenorhabditis Genetics Center (CGC, University of Minnesota). The full list of strains, sources and construction are listed in Key Resource Table.

### METHOD DETAILS

#### Protoarrays

3x-flag-tagged, full length LRRK2, GAK, BAG5, Rab29 and 3x-flag tagged eGFP control proteins were purified as described (Civiero et al., 2012). Six micrograms of each purified 3x-flag-tagged proteins were used to probe Protoarrays, version 5.2 (Thermo Scientific) according to the manufacturer’s instructions with the modification that after 3xflag-tagged protein probing, arrays were probed with monoclonal anti-Flag ® BioM2[C0]Biotin, Clone M2 (Sigma-Aldrich) antibody, followed by probing with Alexa Fluor® 647 streptavidin (Thermo Scientific). Arrays were imaged using an Axon GenePix 4000B fluorescence scanner and images were analyzed using GenePix Pro Software. ProtoArray Prospector software was used to identify significant hit. Binding strength was estimated as Z-scores, i.e., numbers of standard deviations above background fluorescence on the array. Each protein on the array was spotted in duplicate, hence reported values are averaged for both spots. Network visualizations were generated using Cytoscape (Shannon et al., 2003).

#### Cloning

Constructs for 2x-myc- and 3x-flag- tagged LRRK2 full length and domains, GAK, BAG5, RAB29 and GUS have been described previously (Beilina et al., 2014; Taymans et al., 2011). VPS52 full length and domain constructs were PCR amplified with the following primers: VPS52 full length: F_flVPS52 and R_flVPS52; ΔC-VPS52: F_ΔcVPS52 and R_ΔcVPS52; ΔN -VPS52: F_ΔnVPS52 and R_-ΔnVPS52; ΔC/ΔN-VPS52(Sac2): F_SacVPS52 and R_SacVPS52 and cloned into pCR8/GW/TOPO vector (Thermo Scientific). Full-length VPS52 and domains were transferred into the pCMV-2xmyc-DEST, pDEST53-DEST, pAcGFP-DEST and p3xflag-HD-DEST vectors using Gateway recombination technology (Thermo Scientific). The generation of a plasmid encoding Syndetin-GFP was previously described. A plasmid encoding VPS54-GFP was generated by using *Homo sapiens* VPS54 cDNA (Origene, MD) subcloned into pEGFP-N1 (Clontech) vector by Gibson assembly using F-VPS54 and R-VPS54; F_vector and R_vector primers.

#### Cell transfections

Transient transfections of HEK293FT cells and astrocytes were performed using Lipofectamine 2000 and Stem reagents (Thermo Scientific). Primary neuronal cultures were transfected with Calcium phosphate method as described (Jiang and Chen, 2006). For siRNAs, cells were transfected with the SMARTpool ON-TARGETplus scramble or VPS52 or LRRK2 or Rab29 siRNAs using DharmaFECT I transfection reagent for HEK293FT cells or Lipofectamine RNAiMAX (Thermo scientific) transfection reagent for astrocytes.

#### Screen for relocalization of LRRK2 to the trans-Golgi network

Following gene ontology analysis of protoarray hits, proteins categorized as membrane bound organelles were identified. Potential hits in addition to CSNK1A1, ARHGEF7 and NTC were used to order a custom pooled ON-TARGETplus siRNA library (Dharmacon). HEK293FT cells were plated in 96 well plates and reverse transfected with siRNA at a final concentration of 50nM. Twenty-four hours later, cells were transfected with 3xFlag WT LRRK2 together with either 2xmyc WT RAB29 or 2xmyc Q67L RAB29 using Lipofectamine 2000. Thirty hours following plasmid transfection, cells were fixed and stained for M2-Flag, Myc (clone 9E1) and TGN46 before labeling with fluorescent secondary antibodies and Hoechst 33342 (Thermo Scientific). Cells were imaged at 20x objective using a Cellomics VTI Arrayscanner and the percentage of cells with Flag WT LRRK2 and TGN46 positive spots were recorded. For each siRNA, 6 wells containing a minimum of 1000 cells each were analyzed. Samples were compared to NTC siRNA control and ranked.

#### Co-Immunoprecipitation

HEK293FT cells transfected with pCHMWS-3xflag-LRRK2, p3x-flag-HD-DEST-RAB29, p3x-flag-HD-DEST-VPS52, pEGFP-VAMP4 or pEGFP-STX6 plasmids were lysed in IP buffer: 20 mM Tris-HCl pH 7.5, 150 mM NaCl, 1 mM EDTA, 0.3% Triton X-100, 10% Glycerol, 1x Halt phosphatase inhibitor cocktail (Thermo Scientific) and protease inhibitor cocktail (Roche) for 30 minutes on ice. Lysates were centrifuged at 4°C for 10 minutes at 20.000 g and supernatant further cleared by incubation with Easy view Protein G agarose beads (Sigma-Aldrich) for 30 minutes at 4°C. After agarose beads removal by centrifugation, lysates were incubated with anti-flag M2 agarose beads (Sigma-Aldrich) or GFP-Trap agarose beads (ChromoTek) for 1 hour at 4°C on a rotator. Beads were washed six times with IP wash buffer: 20 mM Tris-HCl pH 7.5, 150 mM NaCl, 1 mM EDTA, 0.1% Triton X-100, 10% Glycerol and eluted in 1x kinase buffer (Cell Signaling), containing 150 mM NaCl, 0.02% Triton and 150 ng/μl of 3xflag peptide (Sigma-Aldrich) by shaking for 30 minutes at 4°C. For GFP-Trap beads, elution was performed by incubation of beads with 2X loading buffer (Bio-Rad) for 5 minutes at 95°C. Each co-immunoprecipitation was repeated in 3–4 independent experiments and quantification for the interactions was estimated as a ratio between two immunoprecipitated proteins: endogenous VPS52/3xflag-RAB29, LRRK2/3xflag-VPS52, VPS52/3xflag-LRRK2 or VPS52/ pEGFP-STX6 depending on the experiment. All co-immunoprecipitations in this and other panels are representative of at least two independent experiments.

#### Immunostaining

Primary cultures of astrocytes, cortical neurons or HEK293FT cells were fixed with 4% PFA/1xPBS, blocked with the 5% FBS/1xPBS/ 0.1% Triton for 1 hour at RT. Primary antibodies were diluted in blocking buffer and incubated for 1 hour at RT. After three 5 min washes with 1x PBS, secondary fluorescently labeled antibodies (Thermo Scientific) were diluted in blocking buffer and were incubated for 1 hour at RT. Coverslips were washed three times with 1x PBS, stained for Hoechst nuclear dye (Thermo Scientific) and mounted with ProLong® Gold antifade reagent (Thermo Scientific).

#### SDS-PAGE and Western Blotting

Proteins were resolved on 4%–20% Criterion TGX pre-cast gels (Biorad) and transferred to membranes by semi-dry trans-Blot Turbo transfer system (Biorad). The membranes were blocked with Odyssey Blocking Buffer (Li-Cor Cat #927–40000) and then incubated for 1 hour at RT or overnight at 4°C with the indicated primary antibody. The membranes were washed in TBST (3 × 5 min) followed by incubation for 1 hour at RT with fluorescently conjugated goat anti-mouse or rabbit IR Dye 680 or 800 antibodies (LICOR). The blots were washed in TBST (3 × 5 min) and scanned on an ODYSSEY® CLx (Li-Cor). Quantitation of western blots was performed using Image Studio (Li-Cor).

#### Histological analysis

Each mouse was anesthetized by intraperitoneal injection of ketamine (0.1ml/20mg). The mouse was then transcardially perfused with 25 mL of 1×PBS followed by 50 mL of ice-cold 4% paraformaldehyde in 1 × PBS (pH 7.4). The kidneys were dissected out and post-fixed in 4% paraformalhehyde at 4°C overnight and then transferred to PBS with 30% sucrose for 24 hours. Kidneys were cut into 40 mm sections using a Leica CM1900 cryostat. Sections were washed three times in PBS before blocking for 1 hour in blocking buffer containing 0.3% Triton, 1% BSA, 1% donkey serum in PBS. The same buffer was used for the primary and secondary antibodies. Primary antibodies were incubated overnight at 4°C and Alexa Fluor secondary antibodies incubated at RT for 2 hours. Sections were washed three times, 10 minutes each wash after primary and secondary antibodies. Kidney sections were mounted on glass slides using Prolong Gold mounting media (Life Technologies). Images were obtained using a LSM880 confocal microscope at 63x magnification (Zeiss). Lamp1 structures size was measured using Fiji/ImageJ software.

#### CD8-ciM6PR Retrograde trafficking assay

HEK293FT cells or astrocytes transfected with scramble or VPS52 or LRRK2 or Rab29 siRNAs for 24 hours were transfected with CD8-ciM6PR plasmid for another 24 hours. Then, cells were incubated with anti-mouse CD8 antibodies (BD Biosciences, 20uL per well in 24 well plates) at 4°C for 1 hour, after antibodies were washed and cells were processed for endocytosis at 37°C. Upon CD8 release, ciM6PR gets trafficked from the plasma membrane to the TGN via the endosomal system. Cells were fixed at 0, 15, 30, 45, 60 minutes after endocytosis and stained for anti-mouse AlexaFluor 488 fluorescently labeled antibodies. Cells were also stained for TGN46/TGN38 antibodies and the number of cells with ciM6PR and TGN co-localizations were quantified at each time point.

#### CtxB Retrograde trafficking assay

HEK293FT cells and mouse primary astrocytes were washed twice with PBS and then exposed to CtxB-Alexa 488 (1 μg mL^−1^ in cold medium) for 30 minutes on ice. After incubation, the cells were washed with cold PBS twice and placed in warmed medium at 37°C for the indicated time points. CtxB is therefore transported from the plasma membrane to the TGN through the endosomal system. Cells were then fixed and stained with TGN46 or TGN38 antibodies. Colocalization between CtxB and the TGN was analyzed using the modified Pearson’s Correlation Coefficient (Colocalization threshold, ImageJ).

#### VFG-GPI Anterograde trafficking assay

Post-Golgi trafficking was analyzed as described (Hirata et al., 2015). First, cells were transfected with the VFG-GPI vector and placed at 40°C overnight. Cells were then treated with 100 μg/ml CHX for 2 hours at 20°C, and subsequently VFG-GPI transport to the plasma membrane was chased at 32°C at the indicated times in HEK293FT cells treated with a non-targeting control (NTC) or siRNA against LRRK2, RAB29 or VPS52, or primary astrocytes derived from WT or R1441C mice. To detect VFG-GPI at the plasma membrane, cells were stained against Flag tag in non-permeabilized conditions with the Flag-M2 antibody. Fluorescent intensity was measured using ImageJ (NIH, Bethesda).

#### Preparation of primary cortical neuronal and astrocyte cultures

Primary cortical neuronal and astrocyte cultures were prepared from C57BL/6J newborn (P0) pups. First, dissected mouse cortices were incubated in 1 ml/cortex Basal Medium Eagle (BME) (Sigma-Aldrich), containing 5 U of papain/ (Worthington) for 30 min at 37°C. Five μg of DNase I was added to each cortex preparation, and brain tissue was dissociated into single cells. Cells were washed twice with 10 volumes of BME and counted. Neuronal cultures were plated at 0.6×10^6^ cells/cm^2^ in BME supplemented with B27, N2, 1 mM glutaMAX-I (all from Invitrogen), 0.45% glucose (Sigma-Aldrich), and 2.5 μM cytosine arabinoside (Sigma-Aldrich) for inhibition of glial cell proliferation. Astrocyte cultures were plated at 0.3×10^6^ cells/cm^2^ in DMEM media (Thermo Scientific), supplemented with 10% FBS (Lonza) into tissue culture flasks. For the preparation of purified astrocyte cultures, 7–10 day primary cultures were vigorously shaken to detach microglia and oligodendrocytes. Purity of each culture was assessed with MAP2 for neurons, GFAP for astrocytes, and OLIG2 and IBA1 to exclude oligodendrocytes and microglia. Cultures had 70%–90% of neurons or astrocytes in all experiments.

#### C.elegans strains, RNAi, and dopaminergic neurodegeneration

GFP and G2019S LRRK2 lines were mated to the TU3401 line, and progeny were mated, to generate GFP (DJM001) or G2019S LRRK2 (DJM002) homozygous offspring on a sid-1 mutant background.

For RNAi silencing, concentrated HT115 bacteria containing a pL4440 RNAi vector (empty, vps-50 or vps-54) were seeded onto NGM plates containing 1 mM IPTG. L4 worms were plated on RNAi plates that had been incubated at room temperature for 1 to 2 days. The resulting adults were placed onto new RNAi plates for 6 hours of egg laying and then removed. L4 progeny were placed onto RNAi plates containing 25 μM FuDR to inhibit the development of future generations. Worms were transferred to new RNAi plates containing 25 μM FuDR every 2 to 3 days until they were collected for imaging.

Degeneration of dopaminergic neurons was monitored by examining the expression of GFP driven by the dopaminergic-specific promoter dat-1 as previously described (Yao et al., 2010). Briefly, age-synchronized live worms were mounted on a 4% agarose pad and immobilized using 2 mM levamisole. The four cephalic (CEP) dopaminergic neurons were scored for markers of neurodegeneration using a Leica DM5500B inverted epifluorescence microscope to visualize GFP expression at adult days 9, 11 and 13. Markers of neurodegeneration included absent or fragmented cell soma as well as absent, broken or blebbed axons, and values were expressed as a percent of normal surviving dopaminergic neurons. At each time point, over 40 worms per strain and condition were analyzed in a blinded manner across 4 independent experiments.

#### RNAscope

Adult mouse 16 μm coronal sections were hybridized at 40°C for 2 hours with the RNAscope specific probes for Lrrk2 and Vps52. Tissue pretreatment and hybridization signal amplification were performed using RNAscope 2.5 HD Assay-RED kit (Cat. No 322350). Sections were additionally labeled using DAPI. Images were later acquired using a LSM780 confocal microscope at 10× magnification (Zeiss).

### QUANTIFICATION AND STATISTICAL ANALYSIS

Analyses based on cell counts were either performed in an automated manner with human selection of cell features (for the LRRK2/TGN assay) or by an investigator blinded to treatment/transfection status. Outliers were identified with ROUT with Q set to 1%. Effects of the LRRK2 inhibitor MLi-2 on LRRK2 phosphorylation were normalized to the DMSO treated samples for each phosphorylation site. Statistical analysis for experiments with two treatment groups used Student’s t tests with Welch’s correction for unequal variance. For more than two groups, we used one-way ANOVA or two-way ANOVA where there were two factors in the model, such as time and treatment. Tukey’s post hoc test was used to determine statistical significance for individual comparisons in those cases where the underlying ANOVA was statistically significant and where all groups were compared; Dunnett’s multiple comparison test was used where all groups were compared back to a single control group. Comparisons were considered statistically significant where *, p < 0.05; **, p < 0.01; ***, p < 0.001; ****, p < 0.0001)

## Supplementary Material

1

2

3

4

## Figures and Tables

**Table:** KEY RESOURCES TABLE

REAGENT or RESOURCE	SOURCE	IDENTIFIER
Antibodies		
mouse anti-FLAG	Sigma	clone M2, cat # F3165; RRID:AB_259529
mouse anti-c-Myc	Roche	clone 9E10, cat # 11667149001; RRID:AB_390912
rat anti-c-Myc	Chromotek	clone 9E1, cat # 9e1-100; RRID:AB_2631398
rabbit anti-FLAG	Sigma	cat # F7425; RRID:AB_439687
sheep anti-TGN46 (human specific)	Biorad	cat # AHP500G; RRID:AB_323104
sheep anti-TGN38 (mouse specific)	Biorad	cat # AHP499G; RRID:AB_2203272
rabbit anti-VPS35	GeneTex	cat # GTX108058; RRID:AB_1241448
mouse anti-ciM6PR	Abcam	clone 2G11, cat # ab2733; RRID:AB_2122792
Lamp1 antibodies	DSHB	cat # 1D4B-S; RRID:AB_2134500
rabbit anti-cathepsin D	Calbiochem	cat # 219361; RRID:AB_2087108
rabbit anti-Rab7	Abcam	cat # ab137029; RRID:AB_2629474
rabbit anti-EEA1	Cell Signaling	cat # 3288; RRID:AB_2096811
chicken anti-GFP	Aves Labs	cat # GFP-1020; RRID:AB_10000240
rabbit anti-VPS51	Atlas antibodies	cat # HPA039650; RRID:AB_10673147
rabbit anti-VPS53	Atlas antibodies	cat # HPA024446; RRID:AB_1848680
rabbit anti-VPS52	Bonifacino lab	N/A
Mouse anti-ciM6PR	Abcam	Clone 2G11 cat # ab2733; RRID:AB_2122792
Rabbit anti-ciM6PR	Abcam	Clone EPR6599 cat # ab124767; RRID:AB_10974087
rabbit anti-LRRK2	Abcam	clone MJF2, cat # ab133474; RRID:AB_2713963
rabbit anti-LRRK2 pS1292	Abcam	Clone MJFR-19-7-8, cat # ab203181; N/A
rabbit anti-LRRK2 pS935	Abcam	Clone UDD2 10(12), cat # ab133450; RRID:AB_2732035
mouse anti-GFP	Roche	clones 7.1 and 13.1, cat# 11814460001; RRID:AB_390913
Rabbit anti-EEA1	Cell Signaling	Cat#3288S; RRID:AB_2096811
sheep anti-RAB29	MRC PPU Reagents and Services	cat # RAB7L (1 - 203); N/A
Chemicals, Peptides, and Recombinant Proteins		
MLi2	Merck Pharmaceuticals	N/A
CTxB	Thermo Fisher Scientific	cat # C34775
Experimental Models: Cell Lines		
HEK293FT cells	Thermo Scientific	R70007
Experimental Models: Organisms/Strains		
Lrrk2 ko mice	Huaibin Cai lab	N/A
LacZ-knock in Rab29^tm1a(EUCOMM)Wtsi^	Wellcome Trust Sanger Institute	N/A
Pan Cre mice	Jaxson Laboratory	Stock # 006054
Rab29 KO mice was generated by crossbreeding LacZ-knock in Rab29^tm1a(EUCOMM)Wtsi^ mice with pan Cre expressing mice. Exon 4 was removed, and complete knockout was confirmed by quantitative PCR and western blot.	This paper	N/A
*C.elegans:* strain GFP (cwrls730; Pdat-1::GFP, lin_-_15[+])	Case Western Reserve University	N/A
*C.elegans:* strain G2019S LRRK2 (cwlrs856; Pdat-1::GFP, Pdat-1::LRRK2[G2019S], lin-15[+])	Case Western Reserve University	N/A
*C.elegans:* strain TU3401 line (sid-1[pk3321]; uIs69 [pCFJ90 (myo-2p::mCherry) + unc-119p::sid-1])	Caenorhabditis Genetics Center	N/A
Oligonucleotides		
F_flVPS52 primer: ATGGCCGCCGCTGCGACCAT	This paper	N/A
R_flVPS52 primer: TCAGAAGTTGGGCTTATGCT	This paper	N/A
F_DcVPS52 primer: ATGGAGCAGATGTTGGGA	This paper	N/A
R_DcVPS52 primer: CTAGAATTCCTGTGTCCGAGC	This paper	N/A
F_DnVPS52 primer: ATGGAGCAGATGTTGGGA	This paper	N/A
R_DnVPS52 primer: CTAGAAGTTGGGCTTATGCT	This paper	N/A
F_SacVPS52 primer: ATGGAGCAGATGTTGGGA	This paper	N/A
R_SacVPS52 primer: CTAGAATTCCTGTGTCCGAGC	This paper	N/A
F-VPS54 primer: ATTCTGCAGTCGACG GTACCGCCACCATGGCTTCAAGCCACAGTTC	This paper	N/A
R-VPS54 primer: GTGGCGACCGGTGGATCCCG CCTCTTCTGCTCCCAAATTTC	This paper	N/A
F_vector primer: CGGGATCCACCGGTCGCC	This paper	N/A
R_vector primer: GGTACCGTCGACTGCAGAATTC	This paper	N/A
RNAscope Probe for mouse Lrrk2	Advanced Cell Diagnostics	Cat No. 421551
RNAscope Probe for mouse Vps52	Advanced Cell Diagnostics	Cat No. 505871
Recombinant DNA		
GFP-VAMP4	Thierry Galli	Addgene plasmid # 42313
CD8-ciM6PR	Lei Lu, Nanyang Technological University, Singapore	N/A
GFP-Syntaxin6	Bonifacino Q, NIH	N/A
Syndetin-GFP	Bonifacino Q, NIH	N/A
VSVG-FLAG-GFP-GPI	Morihiss Fujita, Jiangnan University, China.	N/A
Software and Algorithms		
ImageJ	NIH	https://rsbweb.nih.gov/ij/download.html
GraphPad Prism	Graphpad	https://www.graphpad.com/scientific-software/prism/
R version 3.3.1	R	https://www.r-project.org/
Cytoscape	Shannon et al., 2003	https://cytoscape.org/
GenePix Pro Software	Molecular Devises	http://mdc.custhelp.com/app/answers/detail/a_id/18792/~/genepix%C2%AE-pro-7-microarray-acquisition-%26-analysis-software-download-page
ProtoArray Prospector	Thermo Scientific	https://www.thermofisher.com/us/en/home/life-science/protein-biology/protein-assays-analysis/protein-microarrays/technical-resources/data-analysis.html

## Highlights

- The Parkinson’s disease kinase LRRK2 interacts with the GARP complex at the TGN
- LRRK2 stabilizes the binding of GARP to the t-SNARE Syntaxin-6
- LRRK2 regulates retrograde and anterograde transport in a kinase-dependent manner
- Depletion of GARP intensifies neurodegeneration in *C. elegans* expressing G2019S-LRRK2
