# Supplementary material for: The Parkinson’s Disease Protein LRRK2 Interacts with the GARP Complex to Promote Retrograde Transport to the trans-Golgi Network
Source: Cell Rep. Author manuscript; Available in PMC 2020 Jun 25. (PMC7315779; doi:10.1016/j.celrep.2020.107614)
Supplement: 1 [file NIHMS1591489-supplement-1.pdf]

Cell Reports, Volume *31*

Supplemental Information

The Parkinson's Disease Protein LRRK2 Interacts with the GARP Complex to Promote Retrograde Transport to the *trans*-Golgi Network

Alexandra Beilina, Luis Bonet-Ponce, Ravindran Kumaran, Jennifer J. Kordich, Morié Ishida, Adamantios Mamais, Alice Kaganovich, Sara Saez-Atienzar, David C. Gershlick, Dorien A. Roosen, Laura Pellegrini, Vlad Malkov, Matthew J. Fell, Kirsten Harvey, Juan S. Bonifacino, Darren J. Moore, and Mark R. Cookson

Supplementary Figures

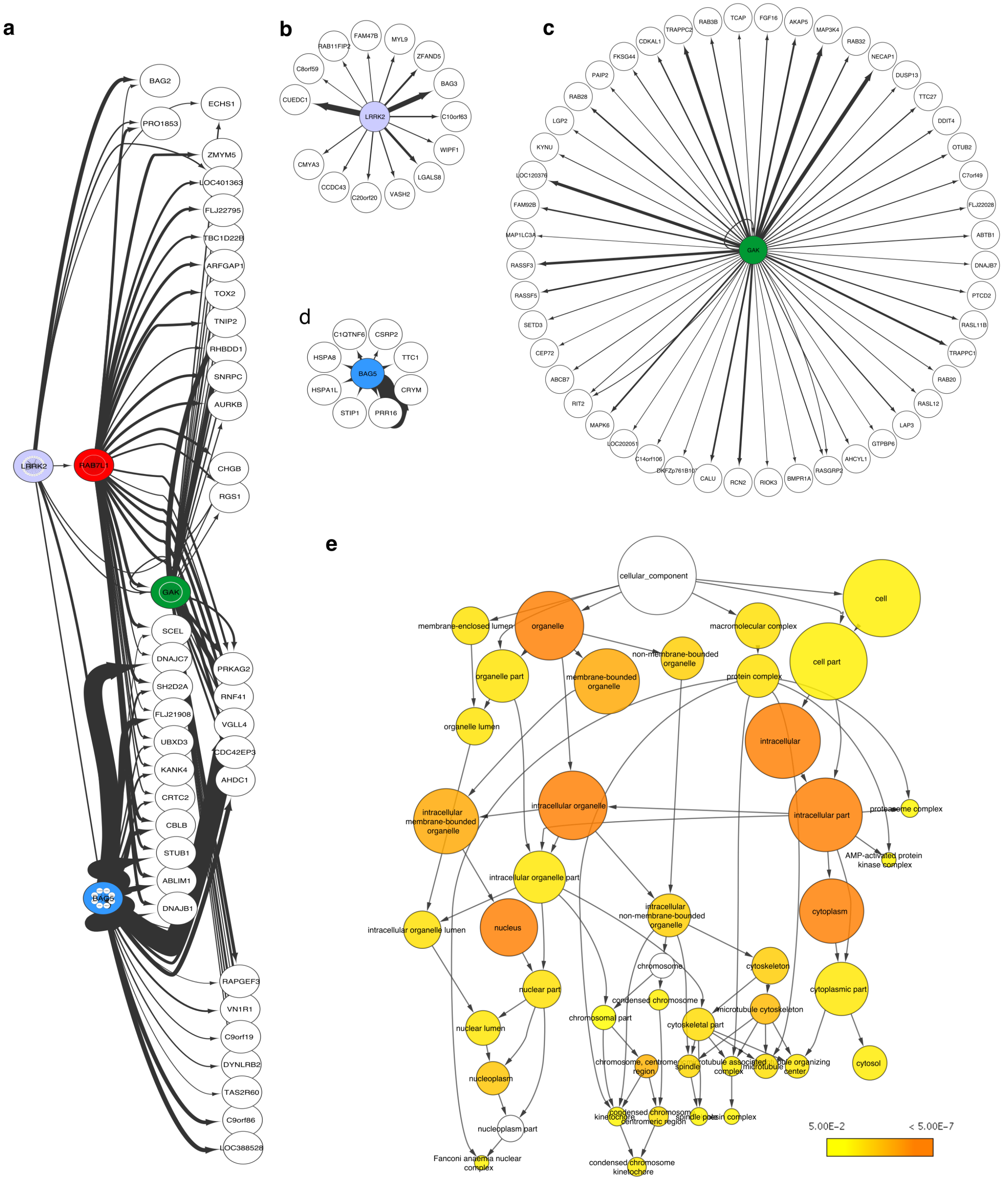

Figure S1. Additional protein network analysis, Related to Figure 1.

(a) The shared candidate protein interaction network. LRRK2 (light purple), RAB29 (red), GAK (green) and BAG5 (blue) protein interaction arrays were visualized looking for shared interactions. Interactions are indicated by arrows, sized to the estimated interaction strength, Z.

(b) Private interactions of LRRK2 not shared with other proteins.

(c) Private interactions of GAK not shared with other proteins.

(d) Private interactions of BAG5 not shared with other proteins.

(e) BinGO analysis of the candidate protein-interaction network using the cellular compartment GO term. Circles are sized by number of candidate interactors in each term and colored by p-value as indicated in the scale on the lower right.

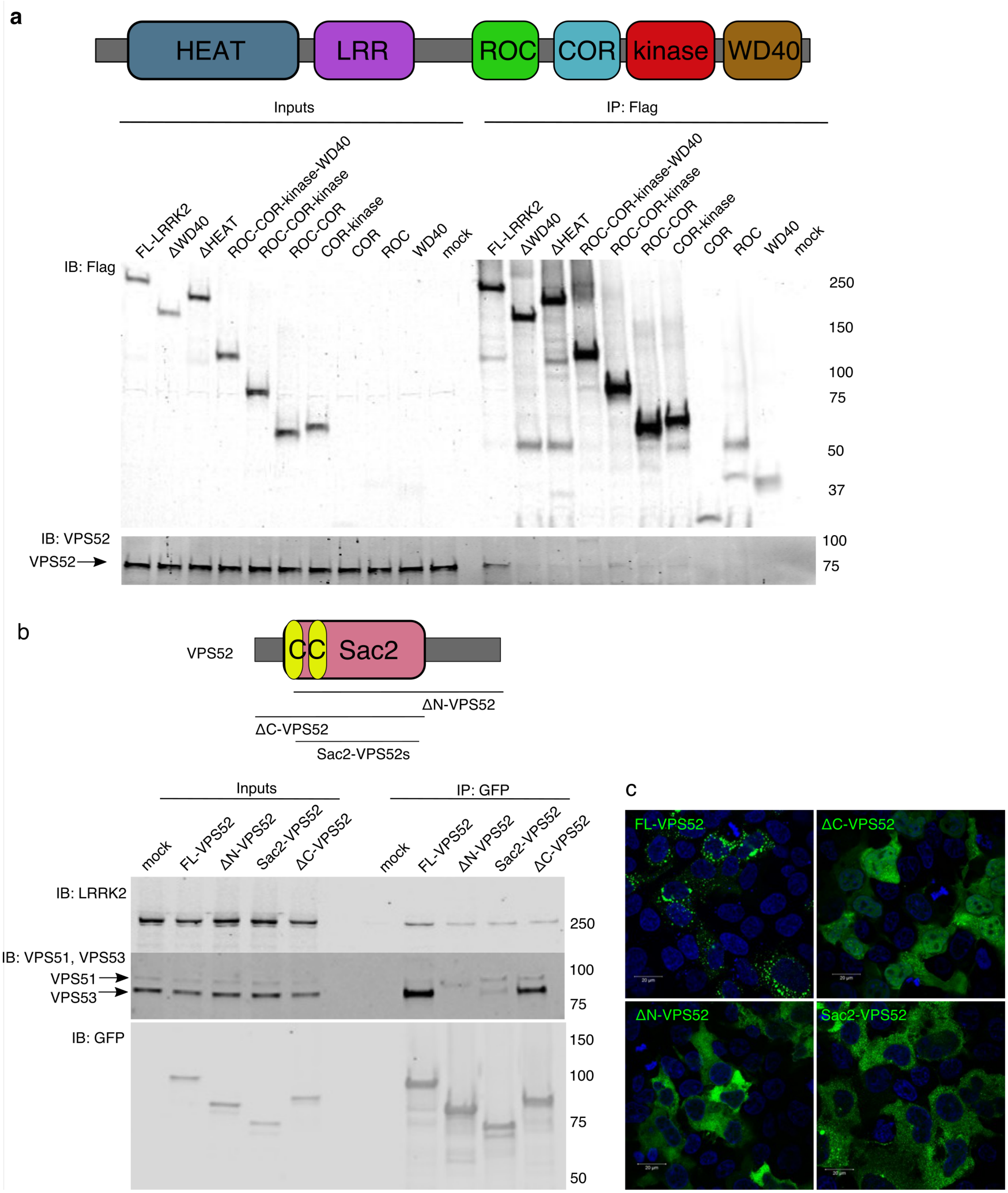

Figure S2. Additional interaction data, Related to Figure 2.

(a) Schematic of the domain structure of LRRK2; LRR = Leucine rich repeats; ROC = Ras of complex proteins; COR = C-terminal of ROC. Flag-tagged versions of the indicated domain constructs, including

full length LRRK2 (FL) were transfected into HEK293FT cells, subjected to immunoprecipitation (IP) with Flag antibody and blotted for endogenous VPS52. Only full-length LRRK2 was able to IP VPS52.

(b) Schematic of the domain structure of VPS52. CC = coiled coil. We made GFP tagged versions of full length and three deletion constructs as indicated. These constructs were transfected into HEK293FT cells, subjected to IP with GFP antibodies and probed for endogenous LRRK2. Full length (FL) VPS52 showed strongest interaction with LRRK2 while the N-terminal region was required for interaction with GARP complex proteins VPS51 and VPS53.

(c) Both N and C terminal regions of VPS52 are required for localization to vesicular structures in HEK293FT cells. Both deletion constructs showed diffuse cytoplasmic staining, with ΔC-VPS52 also being aberrantly present in the nucleus. Scale bar: 20 μm.

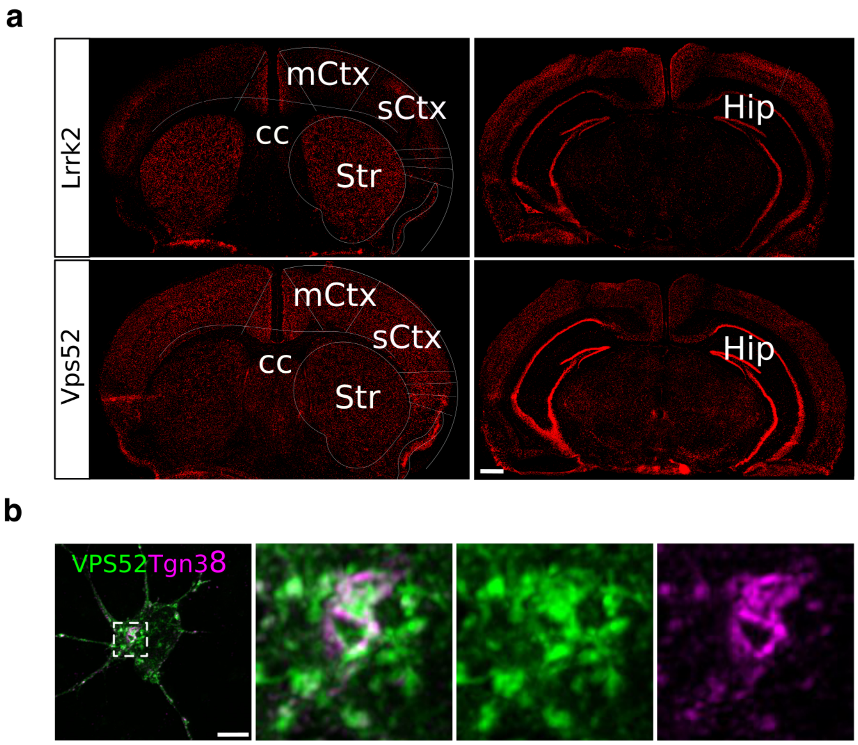

Figure S3. Additional expression data, Related to Figure 3.

(a) Lrrk2 and Vps52 are broadly expressed in the brain. RNAscope in situ hybridization using probes against mouse Lrrk2 (upper panels) or Vps52 (lower panels) at the level of the striatum (left) or hippocampus (right). Both genes are broadly expressed in the brain, with the following areas noted in each figure – cc, corpus callosum; Hip, hippocampus; Str, striatum; mCtx, motor cortex; sCtx, sensorimotor cortex.

(b) Primary mouse neurons transfected with VPS52 (green) and then stained for the TGN marker Tgn38 (magenta). Pictures were taken using the Airyscan module of the confocal microscope. Insert shows VPS52 localization at the TGN. Scale bar: 5 µm.

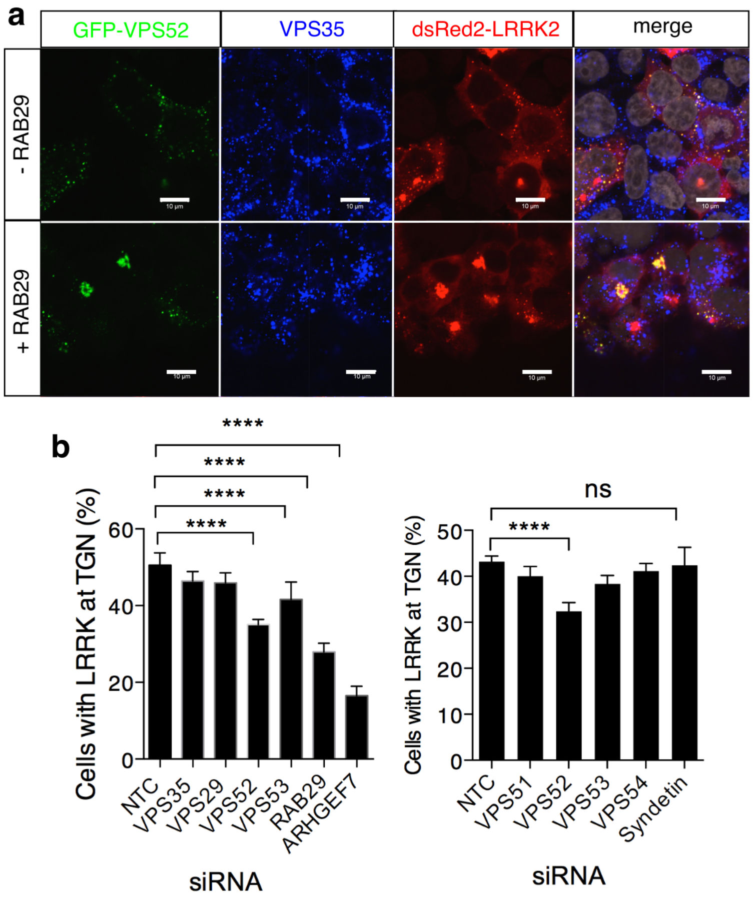

Figure S4. Additional VPS52 dynamics data, Related to Figure 4.

(a) HEK293FT cells were transfected with GFP-tagged VPS52 (green) and dsRed2-tagged LRRK2 (red) either without (upper panels) or with (lower panels) RAB29. Cells were also stained for VPS35 (blue). Note the clustered localization of VPS52 and LRRK2 in the presence of RAB29, with minimal colocalization with VPS35. Scale bar: 10 µm.

(b) HEK293FT cells were transfected with LRRK2 and RAB29, treated with the indicated siRNAs against retromer or GARP components (left graph) or GARP and EARP components (right graph), then stained with TGN markers and relocalization of LRRK2 to the TGN measured using an automated high content imaging system. ARHGEF7 and RAB29 were used as positive controls for this assay as these two endogenous genes were previously shown to be required for recruitment of LRRK2 to the TGN. Treatment with siRNAs had a statistically significant effect on LRRK2 localization to the TGN by one-way ANOVA. Individual siRNAs were compared to non-targeting control (NTC) using Tukey's *post-hoc* test. Error bars indicate the SD between individually treated cultures (n= 6-10 wells per siRNA).

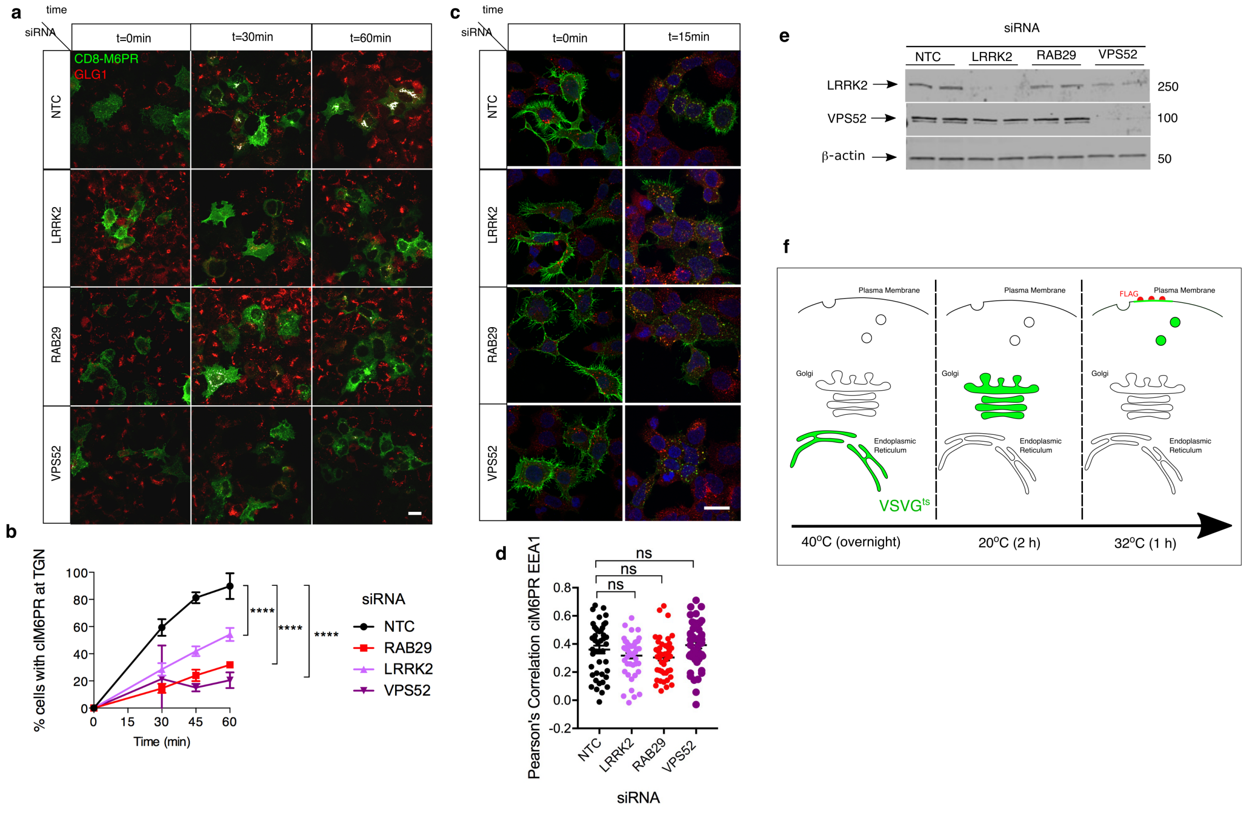

Figure S5. Additional trafficking data, Related to Figure 5.

(a) HEK293FT cells were transfected with CD8-tagged M6PR (green) and treated with siRNA against endogenous LRRK2, RAB29 or VPS52 (NTC= non-targeting control), labelled at 4°C then chased at 37°C for the times indicated times and stained for the TGN marker GLG1. Note that the strong colocalization of CD8-M6PR and GLG1 in the NTC cells (upper right) is not seen with siRNA of LRRK2 and interacting partners. Scale bar: 10 µm.

(b) Quantification of cell uptake of CD8-M6PR in (a). Two-way ANOVA showed a significant effect of both time and siRNA. Multiple comparisons are shown for the siRNAs using Tukey's *post-hoc* test, compared to NTC. Error bars indicate the SD between replicates, n= 3 experiments.

(c) HEK293FT cells were transfected with CD8-tagged M6PR (green) and treated with siRNA against endogenous LRRK2, RAB29 or VPS52 (NTC), labelled at 4°C then chased at 37°C for 15 min and stained for the endosome marker EEA1. No significant differences were observed in any of the groups for colocalization of internalized CD8-M6PR with early endosomal marker EEA1 compared to non-targeting control (NTC). Scale bar: 20 µm.

(d) Quantification of cell uptake of CD8-M6PR in (c). One-way ANOVA showed no significant effect for colocalization of ci-M6PR with EEA1 for all groups compared to non-targeting control. Multiple comparisons were peformed for the siRNAs using Dunnett's *post-hoc* test; *ns*=not significant. Error bars indicate the SEM between replicates, n= 44-48 cells.

(e) Confirmation of knockdown. Parallel duplicate samples from the experiment in (a) were blotted for (from top to bottom), LRRK2, VPS52 and β-actin as a loading control. Representative data from 3 independent experiments.

(f) Schematic diagram depicting the different steps involving the VSVG assay to analyze post-Golgi trafficking. We used a temperature sensitive VSVG strain that accumulates in the ER at 40ºC. After transfection, cells are moved to 20ºC to accumulate VSVG at the TGN and CHX is added here to stop new synthesis at the ER. Later, we start chasing by putting cells at 32ºC for 1h where VSVG gets transported very efficiently to the plasma membrane.

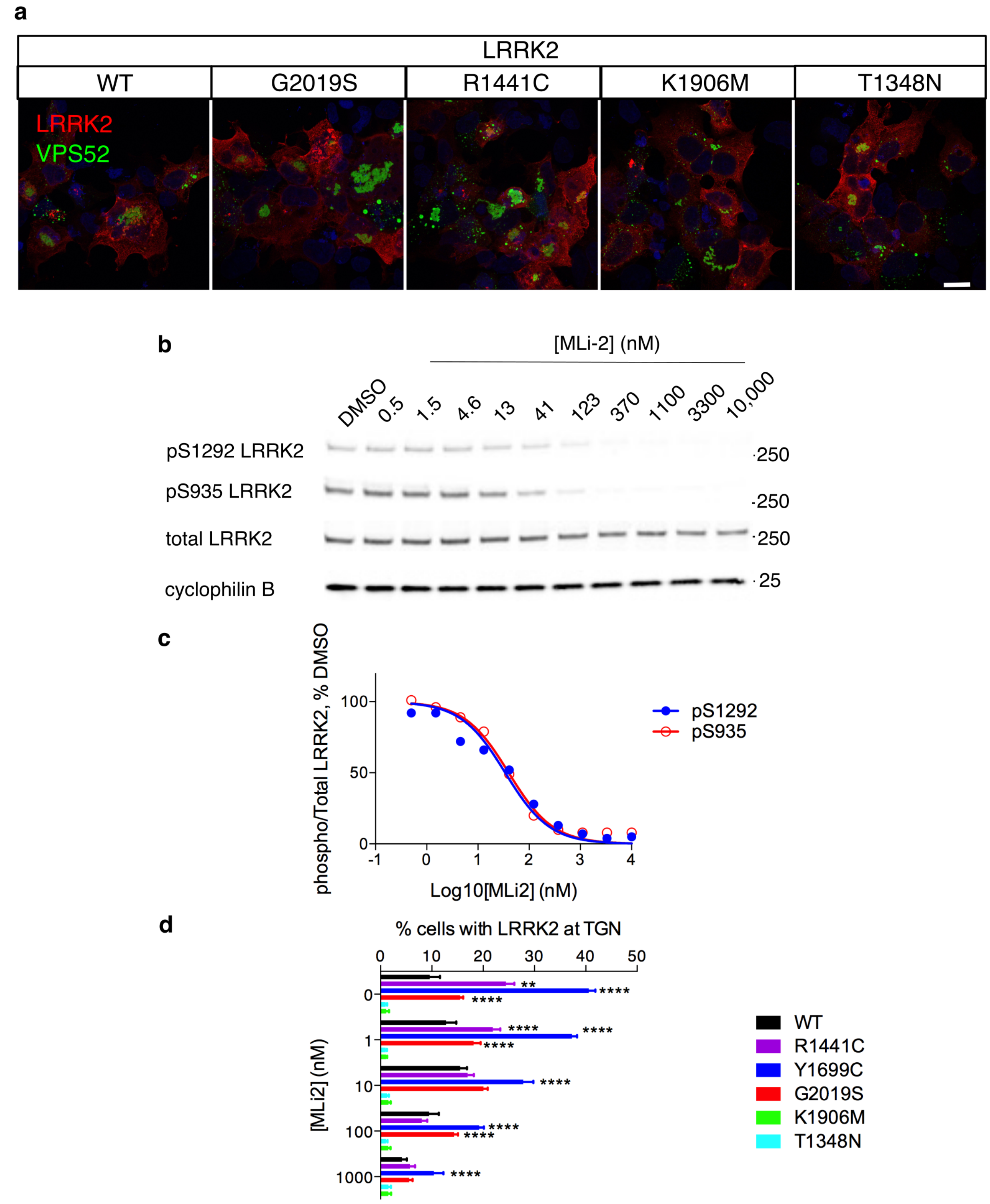

Figure S6. Additional LRRK2 mutation data, Related to Figure 6.

(a) HEK293FT cells were co-transfected with indicated LRRK2 variants (red) and VPS52 (green). Note the presence of large VPS52-positive structures, particularly with mutant LRRK2 but not with kinase dead (K1906M) or GTP-binding deficient (T1348N) LRRK2. Scale bar: 10 μm

(b) HEK293FT cells were treated with the indicated concentrations of the LRRK2 inhibitor MLi2 and protein extracts for the autophosphorylation site pS1292-LRRK2, pS935-LRRK2, total LRRK2 and the loading control cyclophilin B.

(c) Quantification of relative pS1292 (red) and pS935 (blue) LRRK2 to total LRRK2 relative to DMSO treated controls against indicated concentrations of MLi-2. Normalized phosphorylation values were fitted against log10[MLi2] and significant fit and similar IC50 values for both phosphorylation sites (R^2=0.956, IC50=34nM for pS1292; R^2=0.989, IC50=40nM for pS935),

(d) Cells were co-transfected with the indicated LRRK2 constructs and wt RAB29 and the number of cells where LRRK2 was recruited to the TGN counted as a percentage of all transfected cells after 24h treatment with the indicated concentrations of MLi-2. n= 5-6 wells per group. Error bars indicate the SD. Two-way ANOVA identified significant effects of construct MLi2 concentration and a significant interaction term Tukey's *post-hoc* tests indicate significant differences in treated wells relative to DMSO control.
